# Molecular Characteristics of T Cell-Mediated Tumor Killing in Hepatocellular Carcinoma

**DOI:** 10.3389/fimmu.2022.868480

**Published:** 2022-04-29

**Authors:** Wei-feng Hong, Mou-yuan Liu, Li Liang, Yang Zhang, Zong-juan Li, Keqi Han, Shi-suo Du, Yan-jie Chen, Li-heng Ma

**Affiliations:** ^1^Department of Radiation Oncology, Zhongshan Hospital, Fudan University, Shanghai, China; ^2^Department of Medical Imaging, The First Affiliated Hospital of Guangdong Pharmaceutical University, Guangzhou, China; ^3^Department of Medical Oncology, Zhongshan Hospital, Fudan University, Shanghai, China; ^4^Cancer Center, Zhongshan Hospital, Fudan University, Shanghai, China; ^5^Department of Oncology, Luodian Hospital Affiliated to Shanghai University, Shanghai, China; ^6^Department of Gastroenterology, Zhongshan Hospital, Fudan University, Shanghai, China

**Keywords:** hepatocellular carcinoma, T cell-mediated tumor killing, tumor microenvironment, glycolipid metabolism, somatic mutation analysis

## Abstract

**Background:**

Although checkpoint blockade is a promising approach for the treatment of hepatocellular carcinoma (HCC), subsets of patients expected to show a response have not been established. As T cell-mediated tumor killing (TTK) is the fundamental principle of immune checkpoint inhibitor therapy, we established subtypes based on genes related to the sensitivity to TKK and evaluated their prognostic value for HCC immunotherapies.

**Methods:**

Genes regulating the sensitivity of tumor cells to T cell-mediated killing (referred to as GSTTKs) showing differential expression in HCC and correlations with prognosis were identified by high-throughput screening assays. Unsupervised clustering was applied to classify patients with HCC into subtypes based on the GSTTKs. The tumor microenvironment, metabolic properties, and genetic variation were compared among the subgroups. A scoring algorithm based on the prognostic GSTTKs, referred to as the TCscore, was developed, and its clinical and predictive value for the response to immunotherapy were evaluated.

**Results:**

In total, 18 out of 641 GSTTKs simultaneously showed differential expression in HCC and were correlated with prognosis. Based on the 18 GSTTKs, patients were clustered into two subgroups, which reflected distinct TTK patterns in HCC. Tumor-infiltrating immune cells, immune-related gene expression, glycolipid metabolism, somatic mutations, and signaling pathways differed between the two subgroups. The TCscore effectively distinguished between populations with different responses to chemotherapeutics or immunotherapy and overall survival.

**Conclusions:**

TTK patterns played a nonnegligible role in formation of TME diversity and metabolic complexity. Evaluating the TTK patterns of individual tumor will contribute to enhancing our cognition of TME characterization, reflects differences in the functionality of T cells in HCC and guiding more effective therapy strategies.

## Background

Hepatocellular carcinoma (HCC) is the most common primary malignant cancer in the liver. It ranks sixth in morbidity and fourth in mortality among cancers worldwide. The mortality rate of HCC in the United States increased by 43% % between 2000 and 2016, and the average 5-year survival is only 12% in China and 18% worldwide (1, 2). The World Health Organization predicts that in 2030, HCC will account for approximately one million deaths (3).

The single or combined administration of checkpoint inhibitors has shown good efficacy in HCC. In the CheckMate-040 trial, patients with advanced HCC received nivolumab as a single second-line agent and showed a median overall survival (OS) time ranging from 15.6 to 28.6 months, irrespective of the use of sorafenib (4). A clinical trial in China (NCT02989922) involving 220 patients with progressive HCC from 13 centers showed a similar treatment efficacy for camrelizumab and other PD-1 monoclonal antibodies, with an objective response rate of 14.7%, 6-month survival rate of 74.4%, and median OS time of 13.8 months (5).

Although immune checkpoint blockade has become an effective immunotherapeutic approach for HCC, it is very difficult to identify subsets of patients expected to benefit from this strategy before the start of therapy. Immune cells (especially various T cell subtypes), stromal cells, and molecules expressed in the tumor microenvironment (TME) are key determinants of the response to checkpoint blockade. Thorsson et al. classified 33 tumors into six immune subtypes based on data from The Cancer Genome Atlas (TCGA), among which HCC cases were classified as inflammatory or lymphocyte-depleted subtypes (6). In a proteomic study of paired tumor and adjacent normal tissues, 159 cases of hepatitis B virus-related HCC were divided into subtypes with metabolic, proliferative, and tumor immune microenvironment (TIME) disorders, and *PYCR2* and *ADH1A* were found to be differentially expressed and involved in metabolic reprogramming in the subtypes (7). However, the clinical utility of these models for predicting the response to immunotherapy in HCC is limited, and they have not been verified in clinical cohorts.

Using a genome-scale gRNA library knockout screen, Pan et al. revealed that inactivation of *Prbm1*, *Arid2*, and *Brd7*, encoding components of the polybromo and BRG1-associated factors chromatin remodeling complex sensitized melanoma cells to T cell-mediated killing (8). Ru et al. integrated high-throughput screening data including CRISPR/Cas9, shRNA, and RNAi data, and determined that *PTPN2* and *CD47* are genes associated with the sensitivity of tumor cells to T cell-mediated killing (referred to as GSTTKs) (9).

In this study, we utilized a set of identified GSTTKs to distinguish between HCC patient populations with different immunophenotypes and immune cell infiltration characteristics. Additionally, we investigated the metabolic and genomic features of patients and developed a new independent prognostic marker based on T cell-mediated tumor killing (TTK) with the potential to guide individualized treatment of HCC.

## Methods

### Raw Data Retrieval and Preprocessing

A total of 660 HCC samples datasets were procured from three publicly available datasets. Raw RNA sequencing data were standardized by variance-stabilizing transformation (VST) using the DESeq2 package in R, include 349 samples from the Cancer Genome Atlas (https://portal.gdc.cancer.gov/) TCGA-LIHC cohort (10) and 196 samples from the International Cancer Genome Consortium (https://dcc.icgc.org/) ICGC-LIRI-JP cohort (11). The microarray datasets, 115 samples of GSE76427, was downloaded from the Gene Expression Omnibus database (GEO, https://www.ncbi.nlm.nih.gov/geo/) (12). Genes associated with a favorable response to TTK in cancer immunotherapy were obtained from the TISIDB database (http://cis.hku.hk/TISIDB/) and used to established a gene set, referred to as GSTTKs (9).

### Integrated Multi-Omics Analysis

GSTTKs differentially expressed between paracancerous and cancerous tissues were identified using the R package DESeq2 (13), with a false discovery rate < 0.05 and |Log fold change | > 1 as thresholds for significance. GSTTKs significantly associated with OS in HCC were identified by univariate Cox regression using the Survival package in R. A Venn diagram was generated using the VennDiagram package to identify the intersection of differentially expressed GSTTKs and prognostic GSTTKs. Somatic mutations in these genes in patients were described using the maftools R package (14). The copy number variation (CNV) status of each gene was retrieved from TCGA and delineated using GISTIC 2.0 to obtain chromosome information along with the gain or loss status, which was visualized in a circos plot (15). A principal component analysis (PCA) was performed using the PCAtools package in R to determine whether specific GSTTKs in the TCGA-LIHC dataset can distinguish between liver tumor samples and non-tumor samples.

### Recognition of Different TTK Patterns by Unsupervised Clustering

The ConsensusClusterPlus package was employed for unsupervised clustering using the following parameter settings: partitioning around medoid (PAM) based on the center point, merge based on Ward’s distances using the minimum variance method (16). In addition, 1000 times repetitions were conducted for guaranteeing the stability of classification. The proportion of ambiguous clustering (PAC) was used to automatically select the optimal number of subtypes. PCA and tSNE analysis were performed to compare the transcriptional profiles between the different immune subtypes. For the clustering results for the TCGA-LIHC and ICGC-LIRI-JP cohorts, Kaplan–Meier survival curves were plotted and log-rank tests were performed using the survminer and survival packages in R.

### Evaluation of Tumor-Infiltrating Immune Cells

Based on TCGA-LIHC dataset, a single sample gene set enrichment analysis (ssGSEA) was performed to quantitatively detect the relative levels of infiltration of 28 immune cells in the TME (17). The genetic signatures for these 28 immune cells were derived from Charoentong et al. (18). In the ssGSEA, differentially expressed marker genes were employed to evaluate the abundance of immune cells in individual samples. The relative abundance of each type of immune cell was represented as an enrichment score. To further explore the relationship between HCC subtypes and immune cell infiltration in HCC, the Wilcoxon rank sum test was used to analyze the differences in immune cell abundance between HCC subtypes. TIDE (Tumor Immune Dysfunction and Exclusion) algorithm developed by Liu can simulate the two main mechanisms of tumor immune escape: the induction of T cell dysfunction at high cytotoxic T lymphocyte (Cytotoxic T Lymphocytes, CTL) and the prevention of T cell infiltration at low CTL, and predict the response potential of tumor immunotherapy. This algorithm was used to evaluate the LIHC cohort, which is verified with the results of ssGSEA analysis to explore the difference of TME among different TTK patterns of HCC (19). The stromal and immune score was determined using the ESTIMATE package in R to assess the level of immune infiltration. These analyses were performed using the gene set variation analysis (GSVA) (20), ComplexHeatmap and estimate packages in R.

### Annotation and Functional Enrichment Analyses

To evaluate the correlation between molecular subtypes and immune markers, the characteristic signatures related to differentially infiltrating immune cells in the HCC subtypes were collected from previous studies. Data for 148 immunomodulators and inhibitory immune checkpoints, including 41 chemokines, 21 major histocompatibility complex molecules, 18 receptor molecules, 44 immunostimulant molecules, and 24 inhibitory immune checkpoint molecules, were collected from previous studies (18, 21, 22). The Wilcoxon rank sum test was used to analyze the differential expression of these genes between the HCC subtypes. To determine the correlation between molecular subtypes and specific biological processes, annotated gene sets derived from the Kyoto Encyclopedia of Genes and Genomes (KEGG), Molecular Signatures Database (MSigDB), and a study by Mariathasan et al. were used for enrichment analysis and for comparing biological processes among subtypes (20, 23). For typing based on glycolipid metabolism, a glycolysis-cholesterol synthesis axis-related gene set was obtained from Schaeffer et al. (24) and modeling was completed using the R package ConsensusClusterPlus package. GSEA, GSVA, and over-representation analysis (ORA) were performed using the ClusterProfiler (25) and GSVA (20) packages in R.

### Study of Etiology Based on Whole-Genome Data

Somatic mutation information in the mutect2 format for patients with HCC in TCGA-LIHC was converted to the mutation annotation format. The maftools package was used to generate waterfall diagrams to visually represent genes with high mutation frequencies. To investigate differences in the distribution of mutations among the HCC subtypes, differentially mutated genes were identified using *p* < 0.05 as the threshold for significance. Non-negative matrix factorization was carried out to reduce the dimensionality of the mutation matrix for the LIHC dataset, and the optimal number of mutation signatures in different HCC subtypes was identified (26). Thirty tumor mutational signatures that have been reported in COSMIC (https://cancer.sanger.ac.uk/cosmic) were downloaded for comparison with signatures identified by NMF, and mutational signature features of HCC were determined (27). A bar graph of 96 trinucleotide changes was generated for each sample to show the base change profile of each mutation feature. The whole process was performed using the NMF, BSgenome, and MutationalPatterns packages in R.

### Calculation of the TCscore and Assessments of Clinical Significance

The index to represent the TTK level was establish based on the expression data for 18 GSTTKs including risk factors of CA9, SLC1A7, E2F1, RECQL4, AURKA, CENPF, RFPL4B, H2AFZ, KIF11, CDC7, TGIF2LX, MCM10, GRM4 and protective factors of SLC4A10, CAPN11, MYO1B, NR4A3, FGF12. The enrichment score (ES) of gene set that positively or negatively regulated TTK was calculated using single sample gene set enrichment analysis (ssGSEA) in the GSVA package (20), and the normalized differences between the ES of the risk factors minus protective factors was defined as the TTK potential index (TCscore) to computationally dissect the TTK trends of each sample:


TCscore=ESforriskfactors-ESforprotectivefactors

The relationships between the TCscore and clinical characteristics, sensitivity to chemotherapeutics were evaluated. AJCC guidelines recommend the use of antineoplastic drugs such as doxorubicin, mitomycin, vincristine, cisplatin and sorafenib in the treatment of HCC. We predicted the chemotherapy response of each sample to these five drugs based on the GDSC database (the Genomics of Drug Sensitivity in Cancer, https://www.cancerrxgene.org/). The prediction process is realized by pRRophetic (28) packages in R.

### Statistical Analysis

All statistical analyses were conducted using R versions 3.6.3 and 4.0.2. For comparisons of continuous variables between two groups, normally distributed variables were evaluated using independent Student’s *t*-tests, and non-normally distributed data were analyzed using Mann–Whitney U tests (the Wilcoxon rank sum test). The chi-square test or Fisher’s exact test was used for comparisons of categorical variables between two groups. The relationships between gene expression levels were evaluated on the basis of Spearman correlation coefficients. Univariate and multivariate Cox analyses were used to identify independent prognostic factors. Receiver operating characteristic curves were plotted using the SurvivalROC package, and the area under the curve was used to evaluate the accuracy of the TCscore in predicting prognosis. The Rtsne package was used for a t-distributed stochastic neighbor embedding (tSNE) analysis. Two-sided *p* < 0.05 was the threshold for significance.

## Results

### Identification and Characterization of GSTTKs Involved in HCC Progression

**Figure 1:**
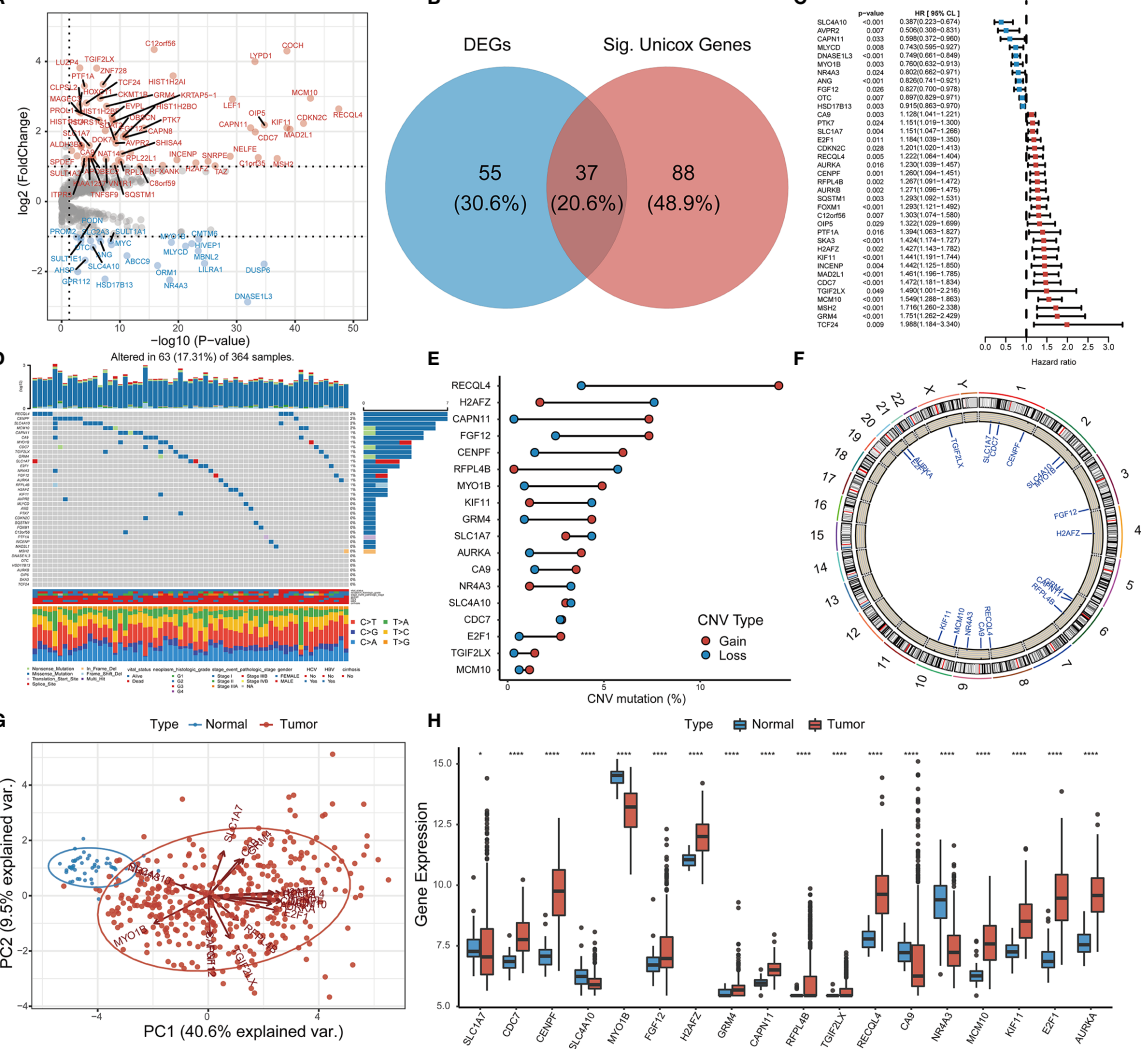
Identification of GSTTKs and detection of mutations in liver hepatocellular carcinoma (HCC). **(A)** Volcano map shows 92 of 641 GSTTKs show differential mRNA expression in HCC based on transcriptome profiling data for patients with HCC from TCGA-LIHC cohort. Red indicates up-regulation and blue indicates down-regulation. **(B)** Venn diagram shows 37 GSTTKs exhibiting both differential expression and prognostic value in HCC. **(C)** Univariate Cox regression analysis of 37 GSTTKs associated with clinical prognosis in HCC. **(D)** Waterfall plot displays the mutational landscape of the 37 GSTTKs along with clinicopathological characteristics. **(E)** Copy number variation (CNV) in 18 GSTTKs in HCC. Deletions, blue dots; Amplifications, red dots. **(F)** CNV locations of 18 GSTTKs are labeled on the chromosome. **(G)** Principal component analysis separates tumor (green) and normal samples (red). **(H)** The 18 GSTTKs are differentially expressed between HCC and normal tissues. Tumor, red; Normal, blue. The upper and lower ends of boxes represent the interquartile range. Lines in the boxes represent median values, and black dots show outliers. Asterisks indicate significance, *p < 0.05; **p < 0.01; ***p < 0.001; ****p < 0.0001; ns, no statistical significance.

Comprehensive analysis of GSTTKs using multi-group data of TCGA-LIHC cohort. The result of difference analysis of transcriptome data shows that 92 of 641 GSTTKs were upregulated or downregulated in HCC, as shown in a volcano map in **Figure 1A** and a heatmap in **Figure S1A**. Univariate Cox regression analysis revealed that 125 out of 641 GSTTKs were related to prognosis in HCC. Taking the intersection of the two sets of genes, 37 GSTTKs simultaneously exhibited differential expression and prognostic value in HCC (**Figure 1B**). The univariate Cox analysis of 37 GSTTKs showed that 11 GSTTKs were protective factors with HR < 1 and 16 GSTTKs were risk factors with HR > 1 for HCC prognosis (**Figure 1C**). According to the genomic data of TCGA-LIHC, the top 10 oncogenic pathways and effects of HCC are shown in **Figure S1B**. The mutational landscape for the 37 GSTTKs is displayed in a waterfall plot in **Figure 1D**. Eighteen out of the 37 GSTTKs had a mutation frequency of >1% and were closely associated with progression or recurrence in HCC. Results of univariate cox regression analysis and differential analysis for 18 GSTTKs were shown in **Table S1**. As shown in **Figure S1C**, the co-occurrence of *CA9* mutations and *MCM10* mutations was significantly overrepresented in HCC. In addition, we detected widespread CNV in these 18 GSTTKs (**Figure 1E**). Copy number gains were most frequent, and *RECQL4*, *CAPN11*, and *FGF12* showed extensive CNV amplification, whereas *H2AFZ* showed a copy number loss. The chromosomal locations of the 18 GSTTKs with CNV are shown in **Figure 1F**. HCC and non-tumor samples could be completely separated by the PCA (**Figure 1G**) based on these 18 GSTTKs with differential mRNA levels (**Figure 1H**), indicating high heterogeneity in the mutation status and expression of GSTTKs between normal and HCC tissues. Thus, GSTTK expression changes may play a crucial role in HCC occurrence and progression.

### TTK Patterns in HCC

**Figure 2:**
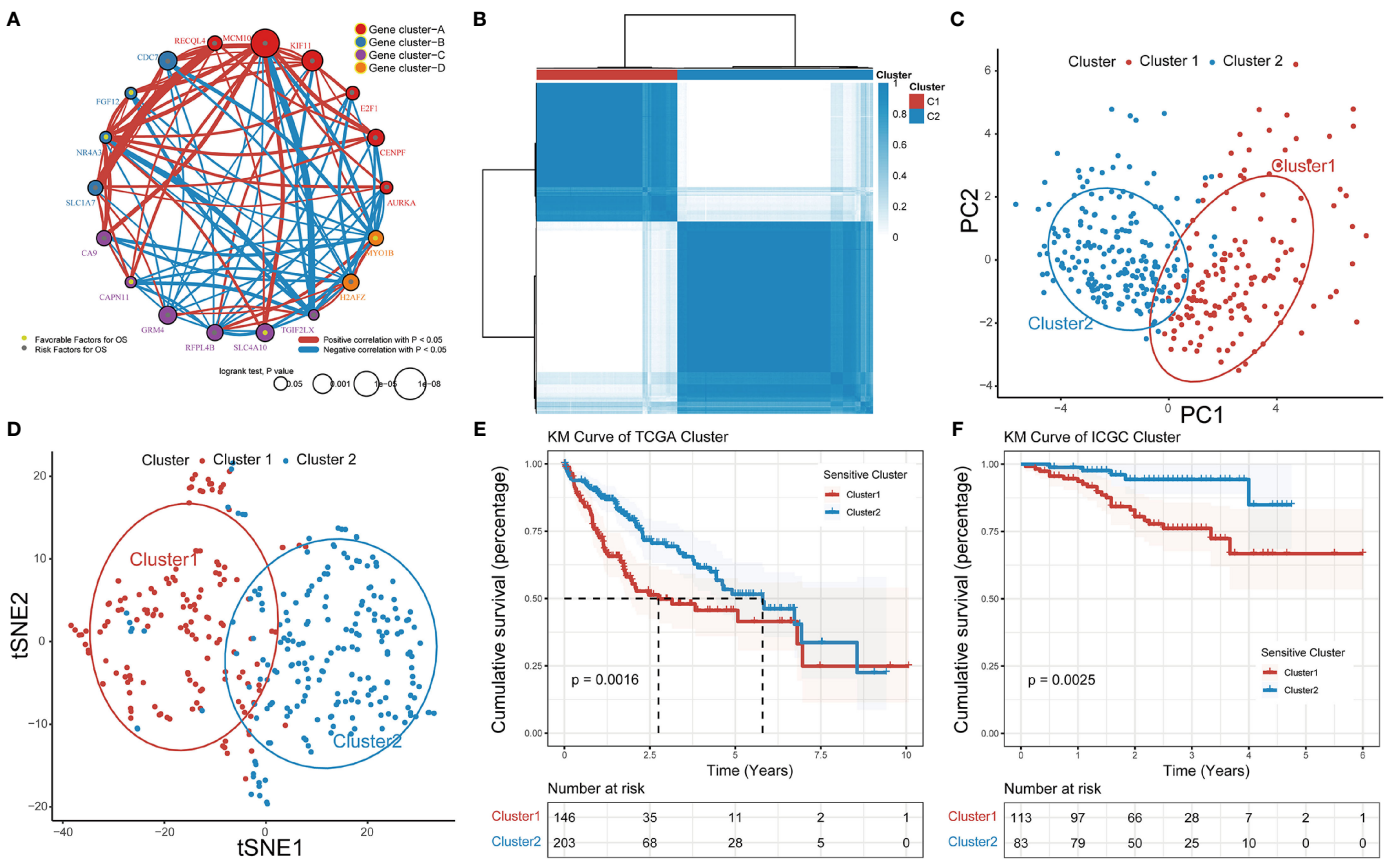
Patterns of TTK and their prognostic value in HCC. **(A)** Interactions among 18 GSTTKs in HCC. The circle size represents the effect of each regulator on prognosis, and comparisons were made using the log-rank test (*p* < 0.05, *p* < 0.001, *p* < 1E-05 and *p* < 1E-08). Green dots in the circle represent risk factors for prognosis; gray dots represent favorable factors for prognosis. The lines linking the regulators show interactions, and the line thickness indicates the strength of the correlation. Positive correlations are marked in red and negative correlations are shown in blue. The regulator clusters A–D are marked in red, blue, purple, and orange, respectively. **(B)** Two patterns of TTK were identified by unsupervised clustering. Cluster1, red; Cluster2, blue. **(C, D)** PCA and tSNE verified the two patterns in HCC. Two subgroups without intersection were identified, indicating that Cluster1 and Cluster2 samples could be clearly distinguished based on GSTTK expression profiles. **(E, F)** Survival analysis indicated that patients assigned to the two clusters had significantly different survival outcomes in TCGA-LIHC and ICGC-LIRI-JP cohorts.

Based on RNA-seq data and clinical data for TCGA-LIHC, we identified four different patterns which show that comprehensive landscape of 18 GSTTKs interactions and their prognostic significance for HCC patients was depicted with the 18 GSTTKs network correlations (**Figure 2A**). The R package of ConsensusClusterPlus was used to classify patients with qualitatively different TTK patterns based on the expression of 18 GSTTKs, and two distinct modification patterns were eventually identified using unsupervised clustering, including 146 cases in Cluster1 and 203 cases in Cluster2 (**Figure 2B**). PCA algorithm and tSNE algorithm are used to evaluate the differences between the two TTK patterns, and it is found that there are significant differences in transcriptional profile among different TTK patterns (**Figures 2C, D**). To verify the stability and applicability of two TTK patterns in HCC, we repeated the unsupervised clustering analysis using LIRI-JP cohort from ICGC (**Figure S2A**) and GSE76427 cohort from GEO (**Figure S2D**); both populations could be well classified into two groups. The PCA (**Figures S2B, E**) and tSNE analysis (**Figures S2C, F**) results corroborated the two distinct patterns of TTK in HCC. Based on TCGA-LIHC expression profiling data, 16 out of 18 GSTTKs in the two clusters were significantly differentially expressed (**Figure S2G**). The clinical prognostic value of TTK patterns in patients with HCC was assessed through a survival analysis. Patients in the two clusters showed a significant difference in survival in both TCGA dataset (*p* = 0.0016, **Figure 2E**) and the ICGC dataset (*p* = 0.0025, **Figure 2F**).

### Mechanisms Underlying the Immunotherapy Response in Patients With Different TTK Subtypes

**Figure 3:**
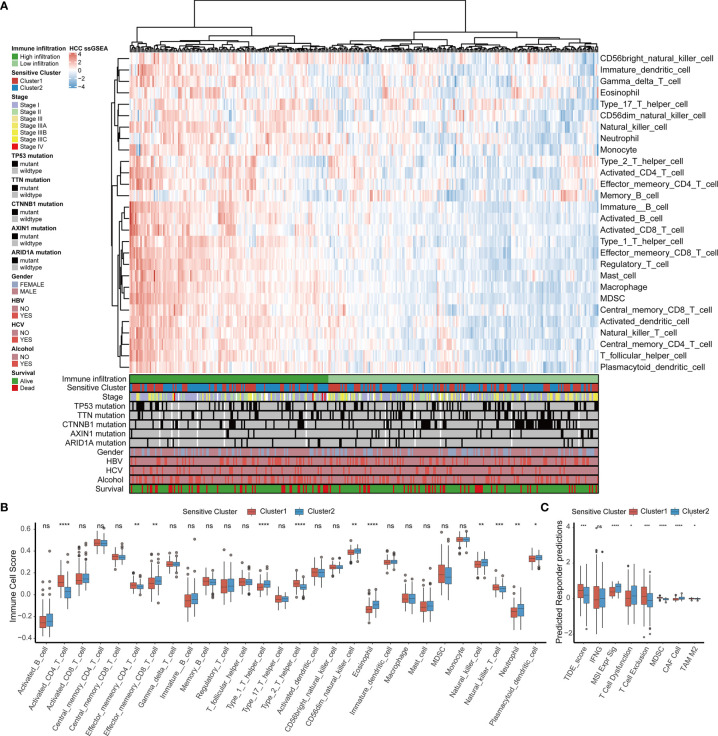
TME immune cell infiltration characteristics and immune components in distinct TTK patterns. **(A)** Heatmap showing the unsupervised clustering of TCGA-LIHC cohort using the ssGSEA score based on 28 immune cell subpopulations. Survival status, alcohol use, hepatitis C or B virus infection, sex, *ARID1A* mutation, *AXIN1* mutation, *CTNNB1* mutation, *TTN* mutation, *TP53* mutation, stage, and sensitive cluster are annotated in the lower panel. **(B)** Relative abundance of each infiltrating cell type that differed between the two clusters. **(C)** Box plots showing the TIDE score for the two clusters in HCC. The upper and lower ends of the boxes indicate the interquartile range. Lines in the boxes indicate median values, and black dots show outliers. *p < 0.05; **p < 0.01; ***p < 0.001; ****p < 0.0001; ns, no statistical significance.

By comparing the infiltrating immune cell composition in the TME of HCC between the two TTK subtypes (**Figure 3A**), we obtained the following key findings. 1) Samples in Cluster 1 mostly showed low immune cell infiltration, whereas samples in Cluster 2 mostly exhibited high immune cell infiltration. 2) The high immune infiltration zone in the heatmap contains immune cells that are established to mediate antitumor immune response (e.g., activated CD8+ T cells, type 1 T helper (Th1) cells, and dendritic cells) and multiple immunosuppressive cells (e.g., bone marrow-derived suppressor cells, regulatory T cells(Treg), immature dendritic cells, and neutrophils), suggesting that there may be a feedback mechanism, that is, TME may promote the recruitment or differentiation of immunosuppressive cells.

In order to determine the specific immune components that cause the difference of TME between Cluster 1 and Cluster 2, the differences of 28 immune cells among different subtypes were calculated. Combined with the results of the survival analysis (**Figure 2E**), samples in Cluster 2 corresponding to favorable survival outcome showed abundant infiltration by effector memory CD8^+^ T cells, Th1 cells, CD56 natural killer cells, eosinophils, natural killer T cells, neutrophils, and plasmacytoid dendritic cells, whereas those in Cluster 1 corresponding to an unfavorable clinical prognosis showed the infiltration of activated CD4^+^ T cells, effector memory CD4^+^ T cells, Th2 cells, and natural killer T cells (**Figure 3B**). A TIDE analysis based on RNA-sequencing data revealed that samples in Cluster 2 had higher scores for T cell dysfunction, microsatellite instability, and tumor-associated fibroblasts than those in Cluster 1, whereas samples in Cluster 1 scored higher for T cell exclusion, myeloid-derived suppressor cells, and tumor-associated M2 macrophages than those in Cluster 2; these findings were generally consistent with the ssGSEA results (**Figure 3C**).

Furthermore, we compared the two clusters with respect to biomarkers of infiltrating immune cells (**Figure S3A**) and molecular markers of the response to immunotherapy, including 41 chemokines, 21 major histocompatibility complex molecules, 18 receptor molecules, 44 immunostimulant molecules, and 24 inhibitory immune checkpoint molecules (**Figure S3B**). In addition, we also evaluated the correlations between the 18 GSTTKs and immune-infiltrating cells (**Figure S4A**) and identified significant correlations between *NR4A3* and *RECQL4* expression and most immune cells. Analysis of the differences in immune-infiltrating cells between the groups with high and low *NR4A3* and *RECQL4* expression were further analyzed (**Figures S4B, C**), and these results indicated that these genes may contribute to the difference between the TTK patterns.

### TTK Patterns and the Metabolic Microenvironment in HCC

**Figure 4:**
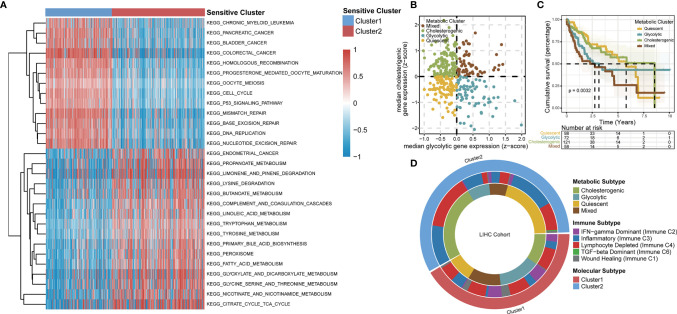
TTK patterns vary with respect to the metabolic microenvironment. **(A)** GSVA enrichment analysis disclosing biological pathway differences between TTK patterns. The heatmap was generated to visualize these biological processes; red represents activated pathways and blue represents inhibited pathways. **(B)** Scatter plot showing median expression levels of co-expressed glycolytic (*x*-axis) and cholesterogenic (*y*-axis) genes in each HCC sample. Metabolic subgroups were assigned on the basis of the relative expression levels of glycolytic and cholesterogenic genes. **(C)** Kaplan–Meier survival analysis of patients with HCC stratified by four metabolic subtypes clustered by glycolytic and cholesterogenic genes. **(D)** Circle diagram shows HCC subtypes for each sample and investigated their degree of overlap with TTK patterns, immune subtypes and metabolic phenotypes.

Based on an enrichment analysis of TCGA-LIHC dataset by GSVA (**Figure 4A**), the two TTK clusters differed significantly with respect to metabolic pathways, suggesting that metabolic alterations as well as the TIME contributed to the distinct TTK patterns. Subsequent ORA (**Figure S5A**) and GSEA (**Figure S5B**) confirmed the difference in metabolic status between the two clusters. Next, we extracted glycolytic and cholesterogenic genes (**Figure S6A**) and used them to classify HCC into four metabolic subtypes: quiescent, glycolytic, cholesterogenic, and mixed (**Figure 4B**). PCA revealed a substantial separation among these four metabolic patterns (**Figure S6B**). The expression levels of genes involved in glycolipid metabolism are presented in **Figure S6C**. We detected significant differences in OS among the four metabolic clusters, with the quiescent and cholesterogenic subtypes being superior to the glycolytic and mixed subtypes (**Figure 4C**, p = 0.0032). This is consistent with the Warburg effect, in which aerobic glycolysis contributes to the aggressive cellular proliferation in malignant tumors. To investigate whether expression patterns across the glycolytic-cholesterogenic axis could underlie the differences between previously established immune subtypes (29), we determined the various HCC subtypes for each sample and investigated their degree of overlap with the metabolic phenotypes (**Table S2**). Quiescent and cholesterogenic subtypes could be classified into Cluster 2, whereas the glycolytic and mixed subtypes were mostly assigned to Cluster 1 or Lymphocyte Depleted Subtype (**Figure 4D**), suggesting that there is a relationship between TTK subtypes and the metabolic microenvironment in HCC. Analysis of the expression levels of the 18 GSTTKs (**Figure S7A**) and tumor-infiltrating cells (**Figure S7B**) according to the metabolic clusters uncovered the relationship between the immune and metabolic microenvironment in HCC.

### Genomic Features and Signaling Pathways Associated With the Two TTK Subtypes of HCC

**Figure 5:**
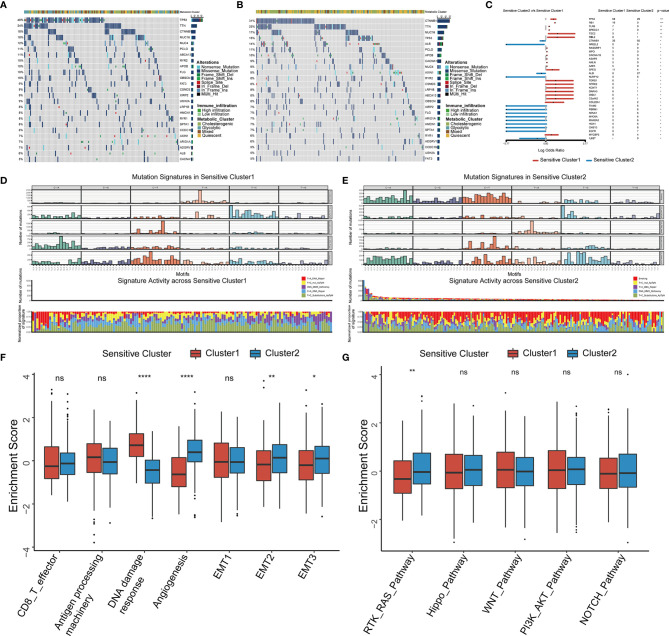
Somatic mutations, mutational signatures, and related signaling pathways for the TTK patterns. **(A, B)** Waterfall plots revealing the somatic mutation distributions of the 25 genes with the highest mutation frequencies in Cluster 1 **(A)** and Cluster 2 **(B)**. **(C)** Forest plot showing differentially mutated genes in the two clusters. **(D, E)** Upper panel, Bayesian NMF was used to identify five mutational signatures in Cluster 1 **(D)** and Cluster 2 **(E)**. The *y*-axis shows the number of mutations of each type in each specific sequence. The middle and lower panels show the total number of mutations associated with five mutational signatures (middle) and relative proportion of mutation types (lower panels) in each TTK pattern. **(F, G)** Differences in signaling pathways involved in patterns of TTK based on gene sets related to stromal-activated pathways and oncogenic signaling cascades. The upper and lower ends of the boxes indicate the interquartile range. Lines in the boxes represent median values, and black dots show outliers. *p < 0.05; **p < 0.01; ***p < 0.001; ****p < 0.0001; ns, no statistical significance.

We analyzed the distribution of somatic mutations in the two clusters using genomic data from TCGA-LIHC datasets (**Figures 5A, B**). Mutations in *CTNNB1*, a common therapy resistance gene in HCC, were predominant in Cluster 1, whereas mutations in *TP53*, a cardinal driver gene of HCC, were predominant in Cluster 2. Comparison of the mutant genes in the two clusters (**Figure 5C**) and revealed that both *TP53* and *RB1* showed the largest difference in mutation frequency between the two clusters. As somatic mutations are the result of multiple mutation processes, including DNA repair defects, and exposure to exogenous or endogenous mutagens, different mutation processes contribute to different combinations of mutation types or characteristics. To comprehensively characterize the landscape of genomic features, we identified five mutational signatures for the two HCC subtypes (**Figure S8**). C > A_DNA_Repair and DNA_MMR_Deficiency predominated in Cluster 1 (**Figure 5D**), whereas Smoking and DNA_MMR_Deficiency were the main patterns in Cluster 2 (**Figure 5E**).

We selected several markers of the stromal TME for a ssGSEA and found that the score for DNA damage response was significantly higher for Cluster 1 than for Cluster 2, whereas the scores for angiogenesis and epithelial interstitial transformation were significantly higher for Cluster 2 than for Cluster 1 (**Figure 5F**). To confirm these results, we calculated the stromal score as well as the ESTIMATE score for cases in TCGA-LIHC using the ESTIMATE algorithm and found significant differences between the two TTK types (**Figures S9A, B**). Moreover, there were significant differences in the stromal and ESTIMATE scores among the four metabolic subtypes (**Figures S9C, D**). Based on the differences in expression patterns and mutation frequencies between the two clusters, we selected genes involved in oncogenic pathways from the MsigDB and KEGG databases for a ssGSEA and found that only the receptor tyrosine kinase (RTK)-RAS pathway differed significantly between the groups (**Figure 5G**). Thus, RTK-RAS is the main pathway mediating the TTK patterns.

### Establishment of the TCscore to Predict the TTK Type in Patients With HCC

**Figure 6:**
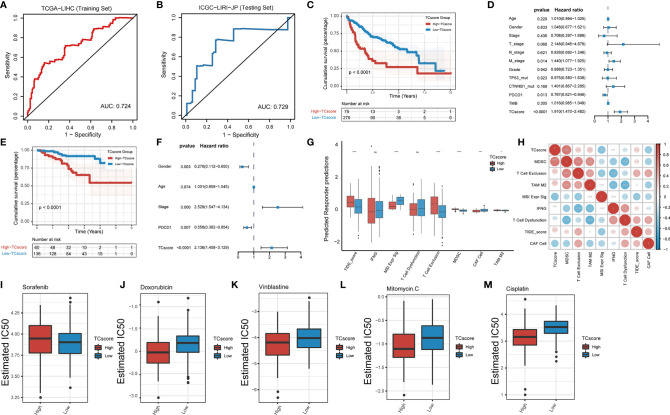
Development of the TCscore based on GSTTKs for the detection of TTK patterns. **(A, B)** time-dependent ROC analysis shows sensitivity and specificity of TCscore in TCGA-LIHC cohort (training set) and ICGC-LIRI-JP cohort (testing set). **(C–F)** evaluation of prognosis for overall survival based on TCscore for cases from the **(C)** TCGA-LIHC cohort and **(E)** ICGC-LIRI-JP cohort. Multivariate Cox regression analyses were performed for patients with HCC from the **(D)** TCGA-LIHC cohort and **(F)** ICGC-LIRI-JP cohort. **(G)** Box plots show the correlation between TIDE score and TCscore in HCC. **(H)** A Spearman correlation analysis indicated that the TCscore is positively related to the TIDE score. **(I–M)** Response to four common chemotherapeutics for high and low TCscore in HCC. *p < 0.05; ***p < 0.001; ****p < 0.0001; ns, no statistical significance.

We developed a scoring system, referred to as the TCscore, to quantify TTK patterns based on the expression levels of the above 18 GSTTKs from TCGA-LIHC. A Spearman correlation analysis of the TCscore, stromal pathway score, and oncogenic pathway score revealed relationships between the TCscore and intertumoral or tumor microenvironment signaling pathways (**Figure S9E**). Most variables were negatively correlated with TCscore, but angiogenesis was most strongly related. TCscore were calculated for patients in the ICGC-LIRI-JP cohort using the formula applied to the TCGA-LIHC cohort to validate the prognostic ability of the GSTTKs signature. The sensitivity and specificity of the TCscore was assessed through time-dependent ROC analysis. The AUC values were 0.724 in TCGA-LIHC cohort (training set) and 0.729 in ICGC-LIRI-JP cohort (testing set), respectively (**Figures 6A, B**). A best threshold value of 0.738 was further selected for classification and Kaplan–Meier and cox regression analysis was performed after classification. Kaplan–Meier curves for OS were plotted according to the optimal cutoff value for TCscore for cases from TCGA-LIHC (*p* < 0.0001, **Figure 6C**) and ICGC-LIRI-JP (*p* < 0.0001, **Figure 6E**). Patients with high TCscore showed a relative shorter survival time than that of patients with low TCscore. The univariate (**Figure S10**) and multivariate cox regression analyses suggested that the TCscore is an independent prognostic factor for patients with HCC in TCGA-LIHC (*p* < 0.0001, hazard ratio (HR) = 1.910, 95% confidence interval (CI): 1.470–2.482, **Figure 6D**) and ICGC-LIRI-JP (*p* < 0.0001, HR = 2.136, 95% CI: 1.458–3.129, **Figure 6F**).

We combined the TCscore with other factors, including the mutation status of known oncogenes (e.g., *TP53*, *ARID1A*, *AXIN1*, *CTNNB1*, and *TTN*) and clinical characteristics (e.g., history of alcoholism, hepatitis B or C virus infection, expression of PDCD1, and tumor mutation burden), and plotted the Kaplan–Meier survival curves based on these parameters for cases from the TCGA database (**Figure S11**). When we also investigated the relationships between the TCscore and clinicopathological characteristics, we found significant correlations of the TCscore with survival, sex, T stage, grade, clinical stage, and TP53 and CTNNB1 mutation statuses (**Figure S12**). To assess the predictive value of the TCscore in immunotherapy, we used the TIDE algorithm to evaluate the associations with the treatment response and found that the differences in the TIDE score in view of the TCscore were similar to the TTK patterns (**Figure 6G**). A Spearman correlation analysis showed that the TCscore was negatively correlated with CAF cell (r = -0.4) and positively correlated with MDSC (r = 0.68, **Figure 6H**).

Using drug information from the GDSC database to calculate the half-maximal inhibitory concentration (IC50) values of common chemotherapeutics for HCC, we found that the IC50 value of sorafenib in the high TCscore group than in the low TCscore group (**Figure 6I**), whereas the IC50 values of four other drugs (doxorubicin, vinblastine, mitomycin, and cisplatin) showed the opposite pattern (**Figures 6J–M**), providing a basis for the selection of chemotherapy drugs when immunotherapy is combined with chemotherapy in clinical practice.

## Discussion

Immunotherapy agents, such as anti-PD1, anti-PD-L1, and anti-CTLA4 antibodies, are increasingly being used in cancer treatment; however, only a subset of patients with HCC benefits from these therapies. Therefore, it is crucially important to characterize the organ-specific TME in HCC and to identify the patient population expected to respond to treatment.

We adopted GSTTKs identified by high-throughput experimental methods to subtype HCC and used unsupervised clustering analysis to further identify TTK patterns. Next, we performed an integrated analysis to evaluate differences in the TIME (e.g., the abundance of tumor-infiltrating cells, molecular markers of immune cells, and immunomodulatory gene expression) and metabolic features (e.g., glycolipid metabolism) between the TTK subtypes. In addition, we evaluated genetic variation, including somatic mutations, mutational signatures, and related signaling pathways, to explore the etiological drivers of the TTK patterns. Finally, we developed a scoring system, the TCscore, based on the TTK types and investigated its clinical and predictive value for the response to immunotherapy.

There is a close connection between immune infiltration and the response to immunotherapy; immune cell dysfunction facilitates the immunosuppressive status in tumors. In this study, we stratified patients with HCC into two stratified according to clinical data from TCGA and validated our findings in an Asian population using data from LIRI-JP in the ICGC. We found that Cluster 2 was dominated by immune cells mediating anti-tumor therapy and that patients in this cluster showed better survival than those in Cluster 1, which was enriched in some of the same immune cells. Chen et al. have reported that tumors with the immune-excluded phenotype also could show abundant infiltration of immune cells trapped in the stroma and excluded from the parenchyma. Conversely, tumors with the immune-inflamed phenotype had greater levels of immune cell infiltration and activation and a better response to immunotherapy (30). Thorsson et al. performed an immunogenomic analysis of cases of 33 cancer types in TCGA and found that HCC can be classified into two types: C3 (inflammatory) and C4 (lymphocyte-depleted) (6). The former type is characterized by the activation of Th1 and Th17 cells and low-to-moderate proliferation of tumor cells, whereas the latter type is characterized by the suppression of Th1 cells and a high response by M2 tumor-associated macrophages. In fact, some pro-inflammatory factors and effector cytokines are released by tumors with the immune-inflamed phenotype, and in some cases, PD-L1 is also expressed, indicating that patients with the immune-inflamed phenotype may show a clear response to immunotherapy (31–33).

We found that *NR4A3* and *RECQL4*, which are involved in the regulation of immunity and metabolism, showed significant associations with most immune-infiltrating cells (34–37). *NR4A3*, a member of the steroid-thyroid hormone-retinoid receptor superfamily, acts as a transcriptional activator by binding to promoter regions to regulate gene expression (38). *NR4A3* binds to *NBRE* to induce the expression of *VCAM1* and *ICAM1* and the adhesion of monocytes, resulting in a tumor necrosis factor-stimulating inflammatory response (39). Li et al. have suggested that *NR4A3* regulates Treg differentiation and maintains the Treg/Th17 balance to improve the symptoms of immune thrombocytopenic purpura (40). Liu et al. have found that *NR4A3* augments glucose uptake in insulin target cells by promoting the translocation of the glucose transporter *SLC2A4* to the cell surface for glucose transport (41). Wang et al. have demonstrated that the suppression of *NR4A3* promotes cell proliferation and disease progression in HCC (42). *RECQL4* is a DNA helicase that modulates chromosome segregation. Wang et al. have revealed that cancer-related *RECQL4* mutations stimulate abnormally high levels of mitochondrial DNA synthesis, resulting in disorders in mitochondrial metabolism. Kumari et al. have reported that *RECQL4* localizes to the mitochondria and dysfunctions in mitochondrial *RECQL4* promote aerobic glycolysis and invasive phenotypes in cells (43). The results of our bioinformatics analyses may guide further experimental studies of the functions and mechanisms of action of these genes (43).

Genes involved in glycolysis-cholesterol synthesis axis have been associated with immune infiltration and prognosis in ovarian, cervical, endometrial, breast, and pancreatic cancers, indicating that there is an interaction between the TME and tumor metabolism (24, 44, 45). Glucose deprivation attenuates the anti-tumor immune response triggered by Cytotoxic T Lymphocytes (CTLs) in glycolytic-dependent tumor cells, whereas checkpoint antagonists, such as anti-PD1 or anti PDL1 antibodies, provide glucose to CTLs by inhibiting glycolysis (46, 47). We hypothesized that this metabolic competition also contributes to TKK and identified four metabolic subtypes in HCC with differences in prognosis, tumor immune-infiltrating cells, GSTTKs expression, and the stromal score.

A recent study revealed that tumor mutations are correlated with the responsiveness or tolerance to immunotherapy (48). Comprehensive genomic analyses have indicated that mutation profiles, including the frequencies of *TP53* and *CTNNB1* mutations, which act as major oncogenic drivers, rather than drug targets in HCC, vary among subtypes (49). In this study, Cluster 1 was characterized by *CTNNB1* mutations and the lymphocyte-depleted phenotype in the TME, suggesting that patients with *CTNNB1* mutations may not be sensitive to immunotherapy. These results were in agreement with those reported by Pinyol et al. (50). Cluster 2 was characterized by a high frequency of *TP53* mutations and the inflammatory phenotype in the TME, suggesting that patients with *TP53* mutations may show favorable responses to immunotherapy. These results were consistent with the previous finding that *TP53* mutations represent the tumor mutational burden in HCC and predict a longer survival time in patients receiving immunotherapy (51). Furthermore, this study revealed other significant indicators of the response to combination therapies. For instance, as *EGFR* and *TSC2* mutations were detected in Clusters 1 and 2, immunotherapy combined with EGFR tyrosine kinase inhibitors (erlotinib or gefitinib) or mTOR inhibitors (sirolimus or everolimus) may be effective for individuals with characteristics of both subtypes.

We identified five mutational signatures in Clusters 1 and 2. Samples in Cluster 1 mainly exhibited two signatures: C > A_DNA_Repair and DNA_MMR_Deficiency, whereas samples in Cluster 2 displayed various signatures, such as Smoking and DNA_MMR_Deficiency. Baecker et al. have reported that tobacco smoking is a risk factor for HCC (52). The difference in DNA damage repair between Clusters 1 and 2 may explain why patients in Cluster 1 showed a worse response to immunotherapy. The mutation pattern in Cluster 1 may contribute to lymphocyte depletion in the TME and the response to immunotherapy. Additional studies are needed to verify these hypotheses.

The mitogen-activated protein kinase pathway and RTKs make up the RKT-RAS-ERK axis, which is crucial for the malignant behavior of common tumors (53). Akalu et al. have reported that TAM receptors, a subfamily of RTKs comprising three members (Tyro3, Axl, and Mer), are an emerging innate immune checkpoint for immune escape and that the inhibition of TAM signaling may promote T cell checkpoint blockade (54). Our results indicated that the RTK-RAS pathway may be the key signaling pathway mediating different TTK modes in HCC. This finding improves our understanding of the biological function and mechanisms of action of T cells in HCC.

We integrated transcriptome data and data for the 18 GSTTKs to establish a new independent quantitative marker, the TCscore, which could be used for individual evaluations of clinicopathological characteristics, sensitivity to chemotherapeutics, and survival outcomes.

This study had some shortcomings. The TTK patterns and TCscore were based on bioinformatics analyses and require validation in a clinical trial with a large sample size. Key GSTTKs and related pathways in TTK patterns, such as *NR4A3*, *RECQL4*, and RTK-RAS signaling, need to be experimentally validated in the future.

## Conclusions

In summary, we identified two TTK patterns in HCC based on GSTTKs, providing insight into T cell activity in HCC. Additionally, we evaluated the mechanism underlying the TTK patterns, including characteristics of the TME, metabolic processes, and multi-omics properties. Finally, the newly developed TCscore, a composite reflection of the TTK patterns of individual tumors, is expected to improve our understanding of the TME and genomic features and to be useful for guiding immunotherapy and combination therapy strategies.

## Data Availability Statement

All data used in this work can be acquired from the GDC portal (https://portal.gdc.cancer.gov/), the International Cancer Genome Consortium (ICGC, https://dcc.icgc.org/) and the Gene-Expression Omnibus under the accession number GSE76427.

## Ethics Statement

The patient data used in this study were acquired from the publicly available datasets with complete informed consent of patients.

## Author Contributions

W-fH and L-hM contributed to conception, design, acquisition, analysis, and interpretation of data. W-fH, Y-jC, and M-yL contributed to the interpretation of result and manuscript preparation. LL, YZ, and Z-jL contributed to the acquisition and interpretation of data. W-fH, KqH, and S-sD revised the manuscript critically. All the authors participated in the discussion and editing of the manuscript. All authors contributed to the article and approved the submitted version.

## Funding

This study was funded by the National Natural Science Foundation of China (No. 81900482), Chinese Society of Clinical Oncology-Youth Innovation Research Fund (Y-young2019-057), and Natural Science Foundation of Shanghai (21ZR1412500).

## Conflict of Interest

The authors declare that the research was conducted in the absence of any commercial or financial relationships that could be construed as a potential conflict of interest.

## Publisher’s Note

All claims expressed in this article are solely those of the authors and do not necessarily represent those of their affiliated organizations, or those of the publisher, the editors and the reviewers. Any product that may be evaluated in this article, or claim that may be made by its manufacturer, is not guaranteed or endorsed by the publisher.

## Supplementary Material

Supplementary Figure 1 Identification of GSTTKs and survey of mutations in these genes in HCC. **(A)** Heatmap displaying 92 GSTTKs differentially expressed in HCC. **(B)** Cellular signaling pathways enriched in mutated genes in HCC. **(C)** Co-occurrence of mutations in pairs of GSTTKs.

Supplementary Figure 2 Patterns of TTK in HCC. **(A–C)** Qualitative classification by unsupervised clustering based on 18 GSTTKs in cases from the LIRI-JP dataset in the ICGC followed by PCA and tSNE analyses. **(D–F)** Qualitative classification by unsupervised clustering based on 18 GSTTKs in cases from the GSE76427 dataset followed by PCA and tSNE analyses. **(G)**. Expression of the 18 GSTTKs in the two TTK patterns in TCGA-LIHC cohort.

Supplementary Figure 3 Immunomodulator biomarkers and involvement in patterns of TTK in HCC. **(A, B)** Heatmaps suggested that signatures of **(A)** nine tumor-associated immune cells and **(B)** immunomodulators as well as immune checkpoints were differently expressed between the two patterns. **p* < 0.05; ***p* < 0.01; ****p* < 0.001.

Supplementary Figure 4 Eighteen GSTTKs are related to tumor immune cell infiltration. **(A–C)** Correlations among 18 GSTTKs and tumor-infiltrating immune cells were studied **(A)**. The expression levels of **(B)** *NR4A3* and **(C)** *RECQL4* were positively associated with various immune cells. **p* < 0.05; ***p* < 0.01; ****p* < 0.001; *****p* < 0.0001.

Supplementary Figure 5 Enrichment analysis indicates a difference in metabolic status between the TTK patterns. **A, B**. **(A)** ORA and **(B)** GSEA revealed differences in metabolic processes between the TTK patterns.

Supplementary Figure 6 Re-clustering of patients according to the glycolipid metabolism patterns in HCC. **(A)** The TCGA-LIHC cohort was re-clustered according to both glycolytic and cholesterogenic genes. **(B)** PCA suggested satisfactory separation among four metabolic patterns. **(C)** A heatmap shows glycolytic and cholesterogenic genes expressed in the four subgroups.

Supplementary Figure 7 Tumor immune microenvironment differed among the four metabolic subgroups. **(A)** Eighteen GSTTKs were generally differentially expressed among the four metabolic subtypes. **(B)** Most tumor immune cells showed differential infiltration among the four metabolic subtypes. **p* < 0.05; ***p* < 0.01; ****p* < 0.001; *****p* < 0.0001.

Supplementary Figure 8 Comprehensive genomic analyses revealed distinct mutational signatures between the TTK patterns. **(A, C)** Bayesian non-negative matrix factorization was used to determine the optimal number of mutational signatures. **(B, D)** Based on 30 mutation features summarized in the COSMIC database, five signatures for the two clusters were annotated.

Supplementary Figure 9 Investigation of the stromal TME in HCC. **(A–D)** The stromal tumor microenvironments **(A, B)** for the two clusters and **(C, D)** four metabolic subtypes were assessed based on the stromal score and ESTIMATE score. **e** A Spearman correlation analysis was performed to evaluate relationships among the TCscore, stromal pathway score, and oncogenic pathway score.

Supplementary Figure 10 The TCscore predicts prognoses in HCC. **(A, B)** Univariate cox regression analysis suggests that the TCscore is associated with a poor prognosis in patients in the TCGA **(A)** and ICGC **(B)** datasets.

Supplementary Figure 11 Survival analysis using the TCscore combined with other factors. **(A–J)**. Kaplan–Meier survival analyses were performed based on the TCscore plus the following factors: mutation statuses of **(A)** *TP53*, **(B)** *ARID1A*, **(C)** *AXIN1*, **(D)** *CTNNB1*, and **(E)** *TTN*, **(F)** history of alcoholism, **(G)** hepatitis B virus infection, **(H)** hepatitis C virus infection, **(I)** PDCD1 expression, and **(J)** tumor mutation burden.

Supplementary Figure 12 The TCscore was related to clinicopathological characteristics of patients with HCC. **(A–G)** Relationships between the TCscore and clinicopathological characteristics, such as **(A)** survival status, **(B)** gender, **(C)** TP53 mutations, **(D)** CTNNB mutations, **(E)** T stage, **(F)** grade, and **(G)** TNM stage, were investigated.

## Abbreviations

CNV, copy number variation; ES, enrichment score; GEO, Gene Expression Omnibus; GSEA, gene set enrichment analysis; GSTTKs, genes sensitive to T cell-mediated tumor killing; GSVA, gene set variation analysis; HCC, hepatocellular carcinoma; HR, hazard ratio; IC50, half-maximal inhibitory concentration; ICGC, International Cancer Genome Consortium; KEGG: Kyoto Encyclopedia of Genes and Gnomes, LIHC, liver hepatocellular carcinoma; MSigDB, Molecular Signatures Database; ORA, over-representation analysis; OS, overall survival; PCA, principal component analysis; RTK, receptor tyrosine kinase; SD, stable disease; ssGSEA, single sample gene set enrichment analysis; TCGA, The Cancer Genome Atlas; Th, T helper; TIME, tumor immune microenvironment; TME, tumor microenvironment; tSNE, t-distributed stochastic neighbor embedding; TTK, T cell-mediated tumor killing.
