# Supplementary figures and images for: Molecular Characteristics of T Cell-Mediated Tumor Killing in Hepatocellular Carcinoma
Source: Front Immunol. 2022 Apr 29;13:868480. doi: 10.3389/fimmu.2022.868480 (PMC9100886; doi:10.3389/fimmu.2022.868480)

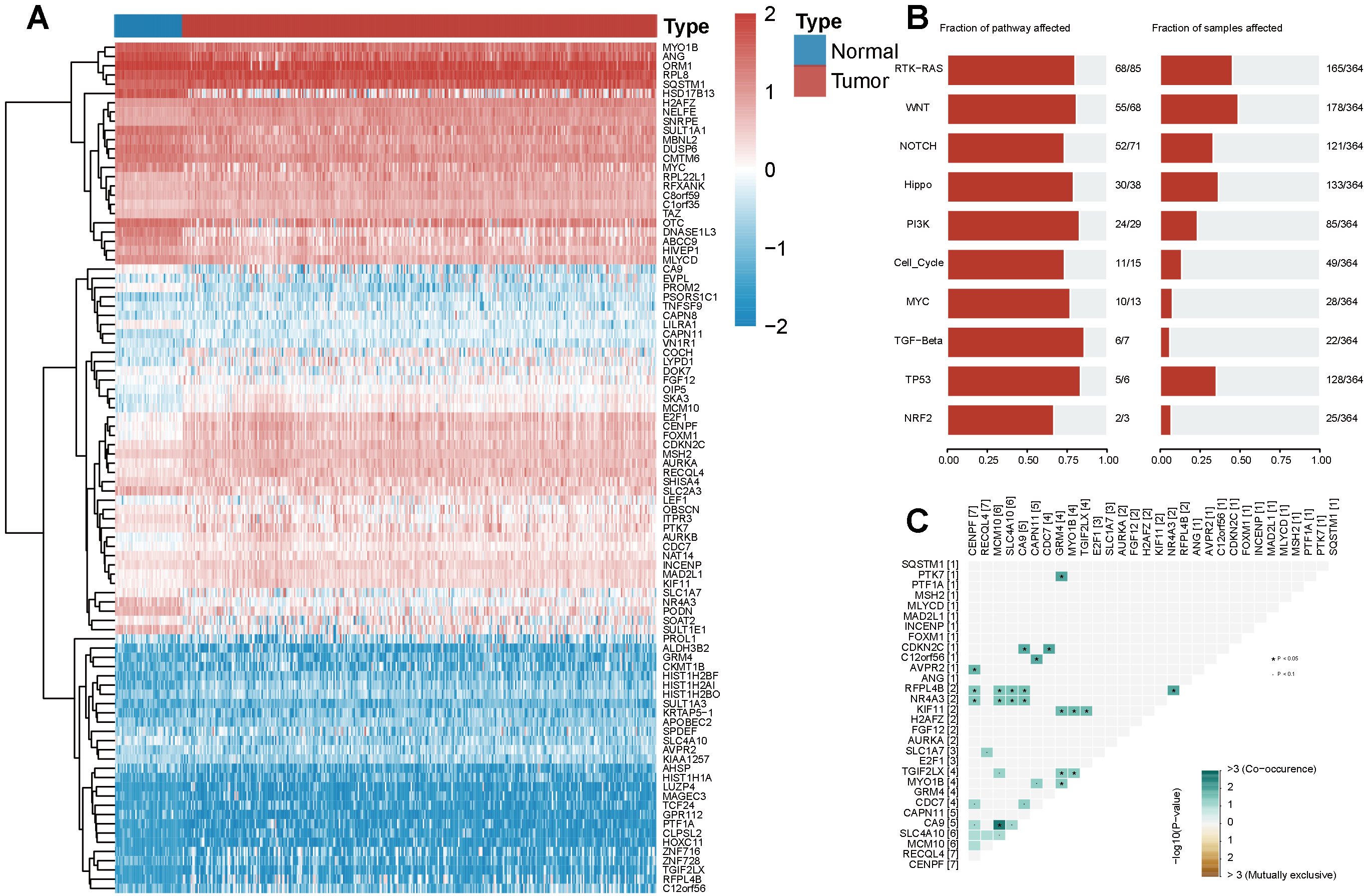

Supplement: Supplementary Figure 1 — Identification of GSTTKs and survey of mutations in these genes in HCC. (A) Heatmap displaying 92 GSTTKs differentially expressed in HCC. (B) Cellular signaling pathways enriched in mutated genes in HCC. (C) Co-occurrence of mutations in pairs of GSTTKs. [file Image_1.tif]

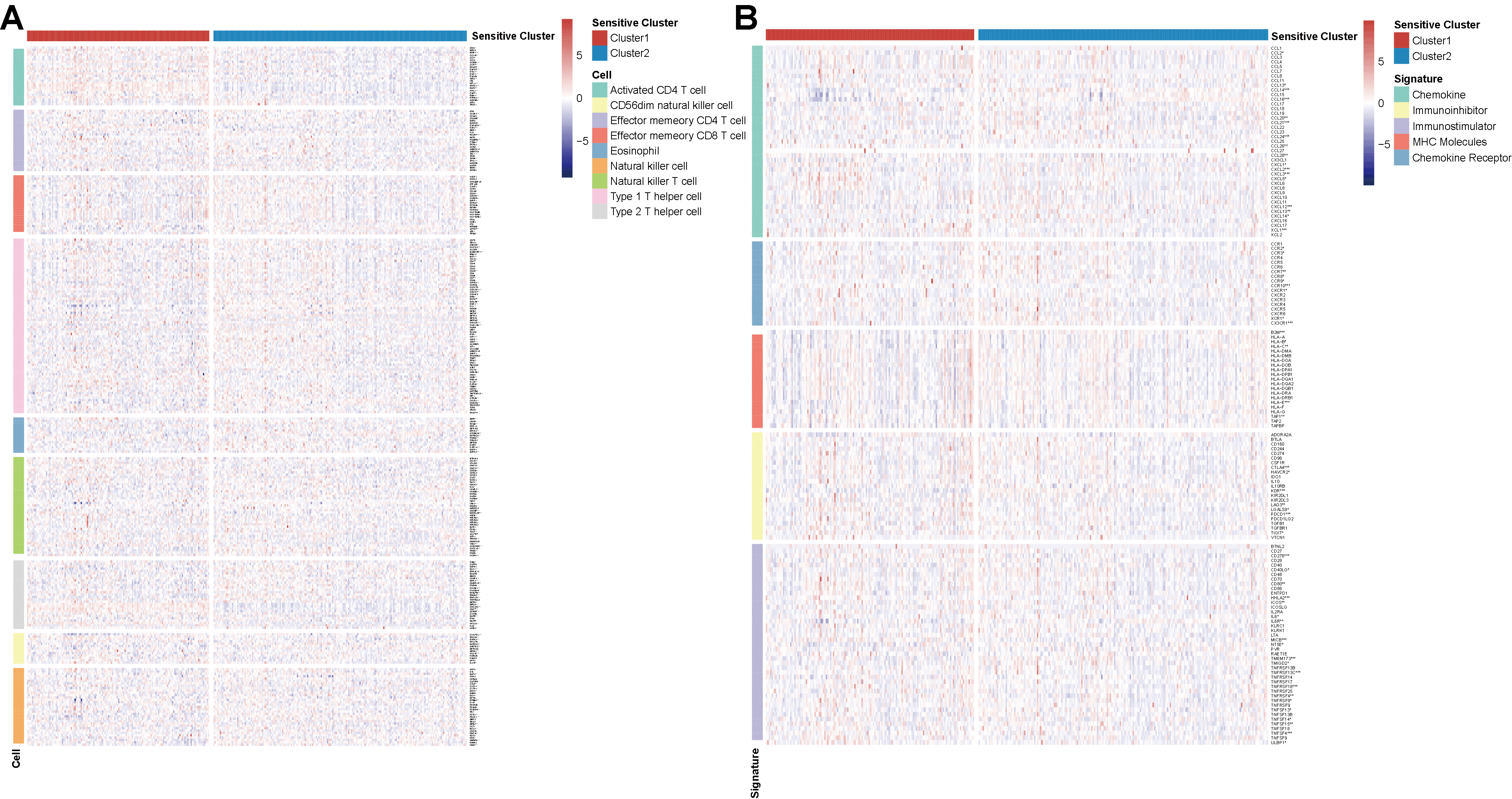

Supplement: Supplementary Figure 3 — Immunomodulator biomarkers and involvement in patterns of TTK in HCC. (A, B) Heatmaps suggested that signatures of (A) nine tumor-associated immune cells and (B) immunomodulators as well as immune checkpoints were differently expressed between the two patterns. *p < 0.05; **p < 0.01; ***p < 0.001. [file Image_3.tif]

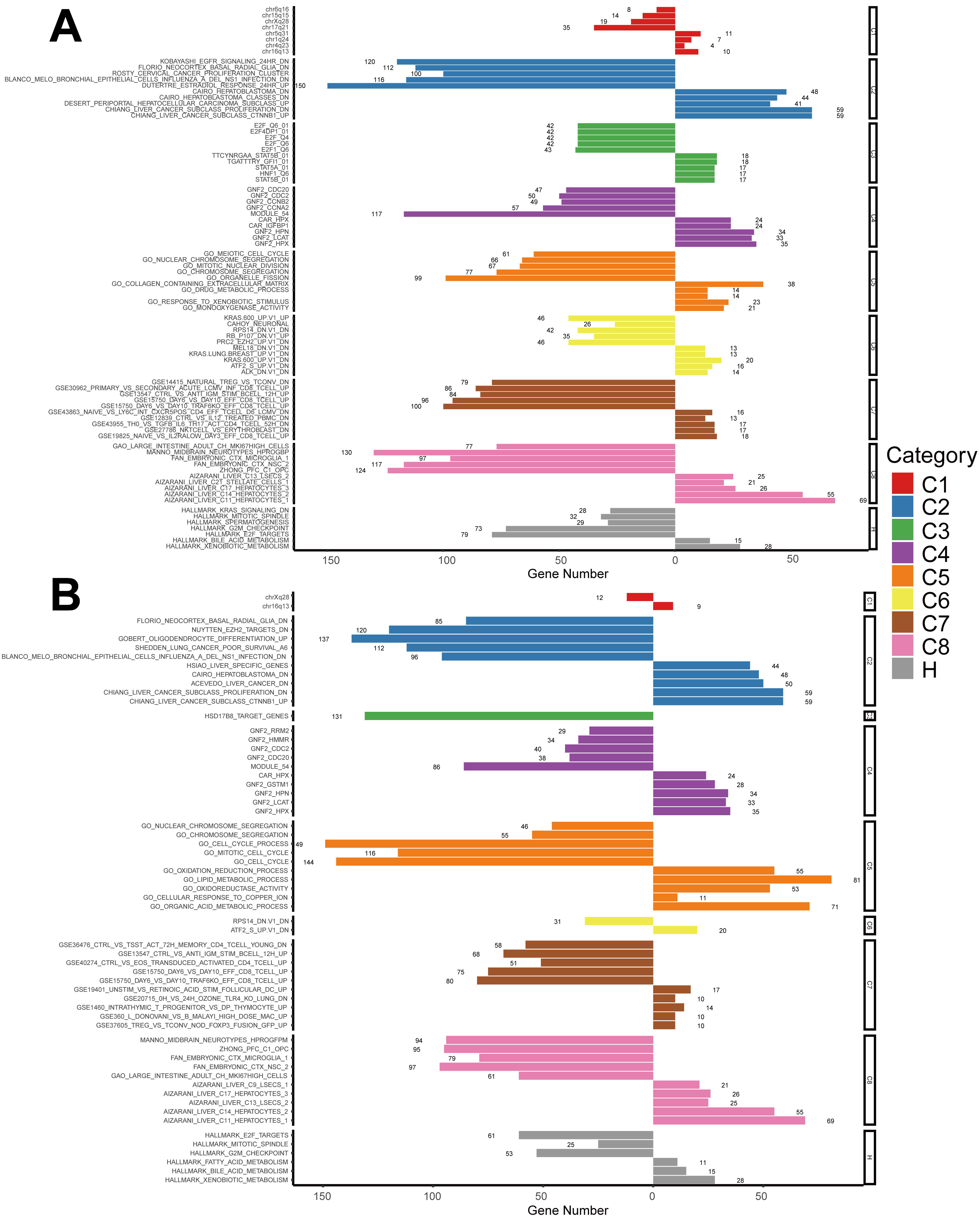

Supplement: Supplementary Figure 5 — Enrichment analysis indicates a difference in metabolic status between the TTK patterns. A, B. (A) ORA and (B) GSEA revealed differences in metabolic processes between the TTK patterns. [file Image_5.tif]

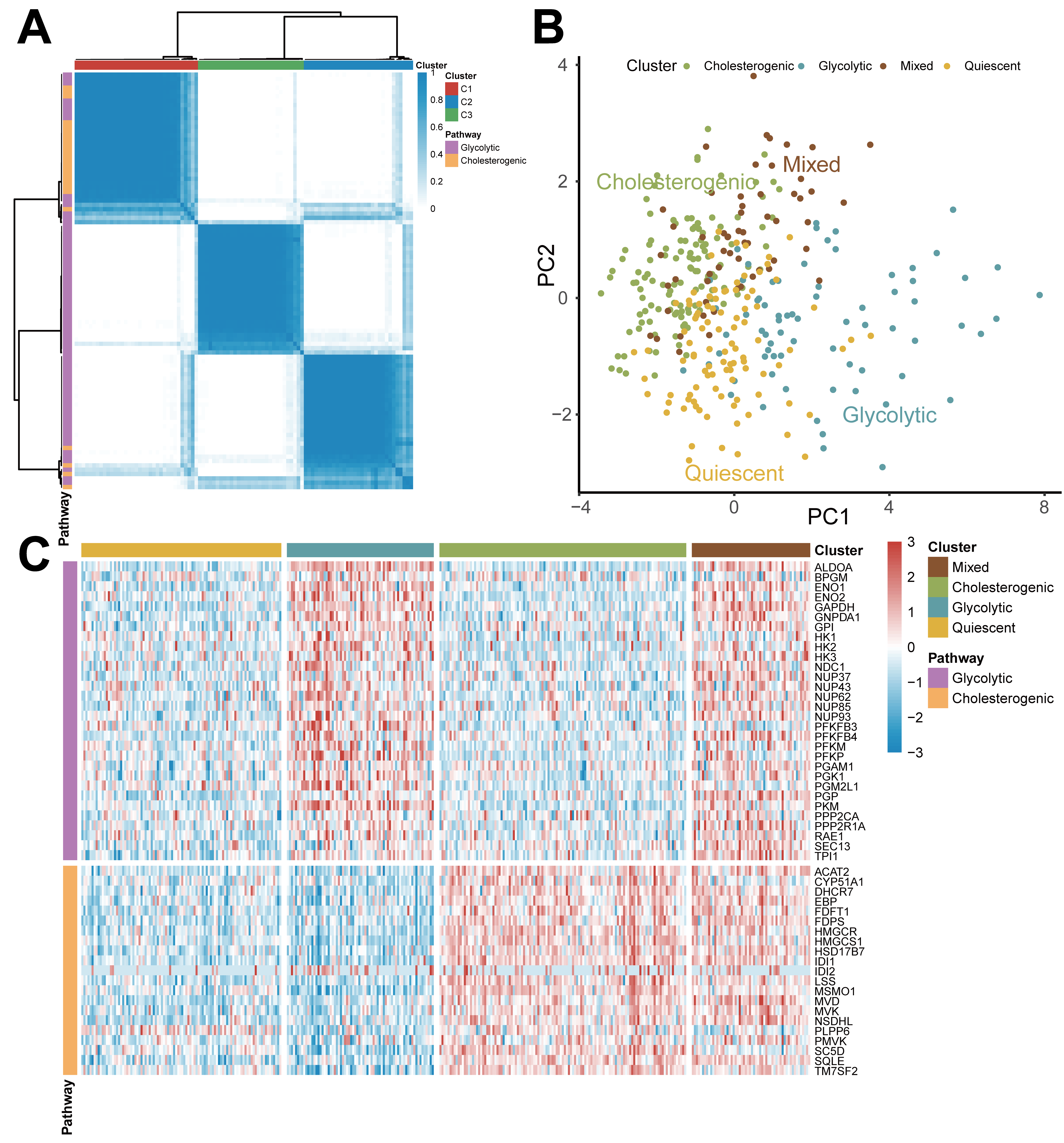

Supplement: Supplementary Figure 6 — Re-clustering of patients according to the glycolipid metabolism patterns in HCC. (A) The TCGA-LIHC cohort was re-clustered according to both glycolytic and cholesterogenic genes. (B) PCA suggested satisfactory separation among four metabolic patterns. (C) A heatmap shows glycolytic and cholesterogenic genes expressed in the four subgroups. [file Image_6.tif]

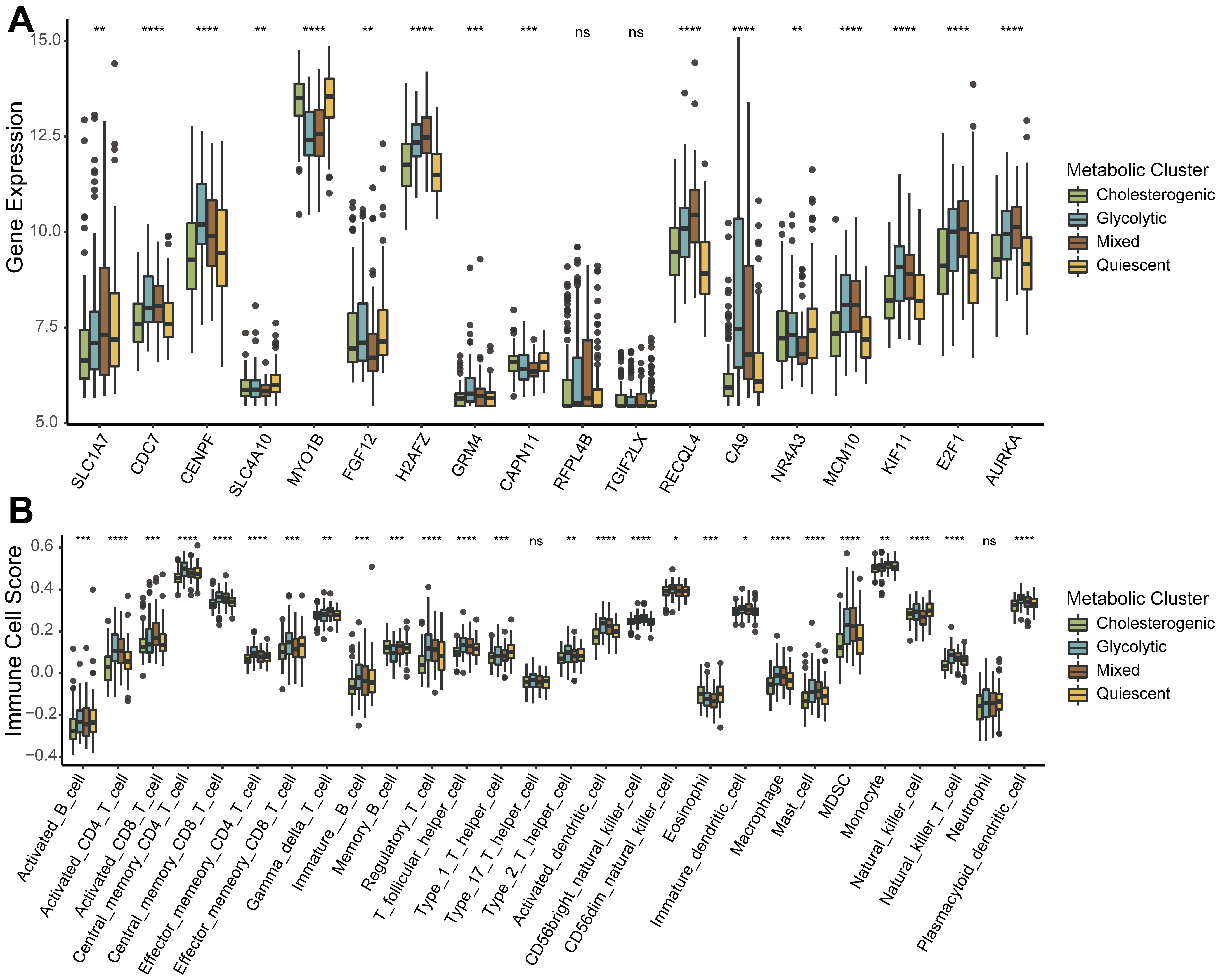

Supplement: Supplementary Figure 7 — Tumor immune microenvironment differed among the four metabolic subgroups. (A) Eighteen GSTTKs were generally differentially expressed among the four metabolic subtypes. (B) Most tumor immune cells showed differential infiltration among the four metabolic subtypes. *p < 0.05; **p < 0.01; ***p < 0.001; ****p < 0.0001. [file Image_7.tif]

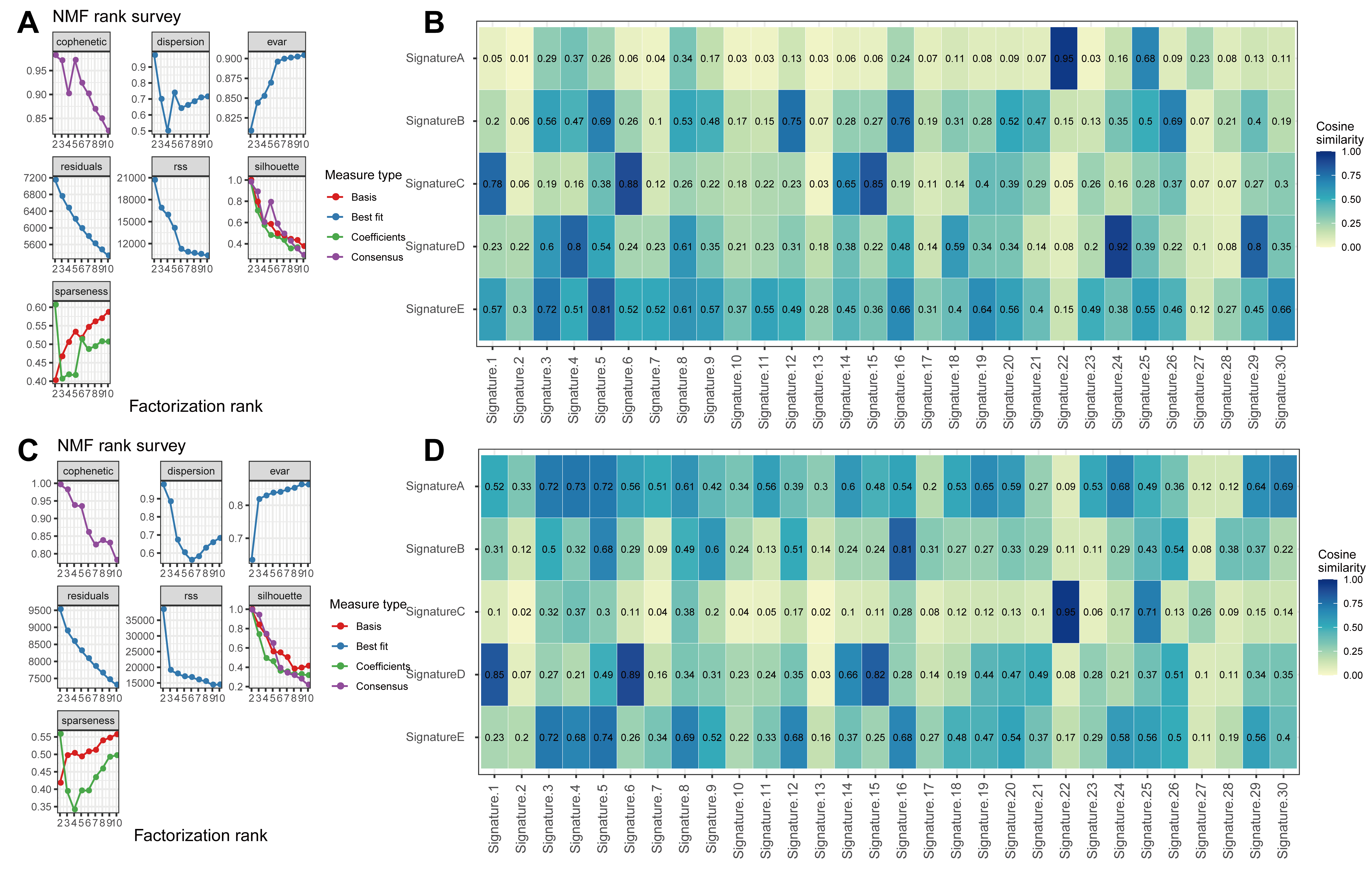

Supplement: Supplementary Figure 8 — Comprehensive genomic analyses revealed distinct mutational signatures between the TTK patterns. (A, C) Bayesian non-negative matrix factorization was used to determine the optimal number of mutational signatures. (B, D) Based on 30 mutation features summarized in the COSMIC database, five signatures for the two clusters were annotated. [file Image_8.tif]

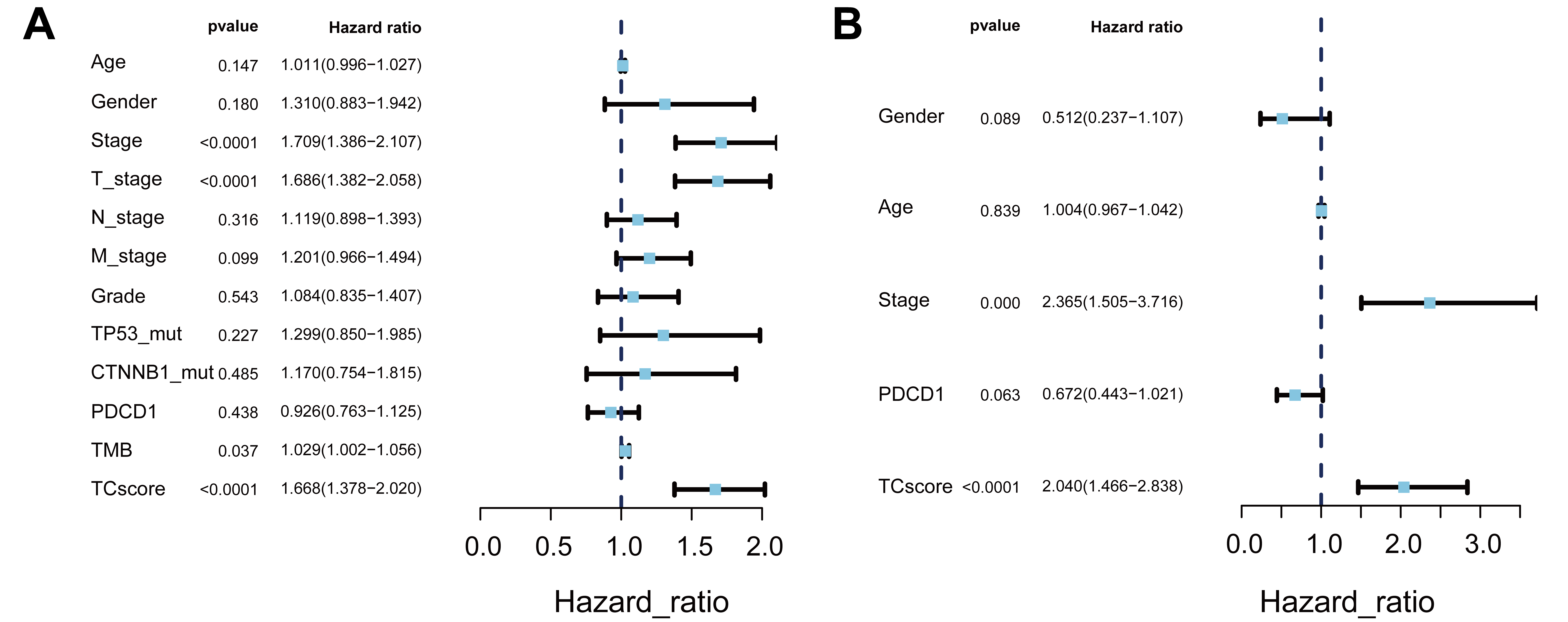

Supplement: Supplementary Figure 10 — The TCscore predicts prognoses in HCC. (A, B) Univariate cox regression analysis suggests that the TCscore is associated with a poor prognosis in patients in the TCGA (A) and ICGC (B) datasets. [file Image_10.tif]

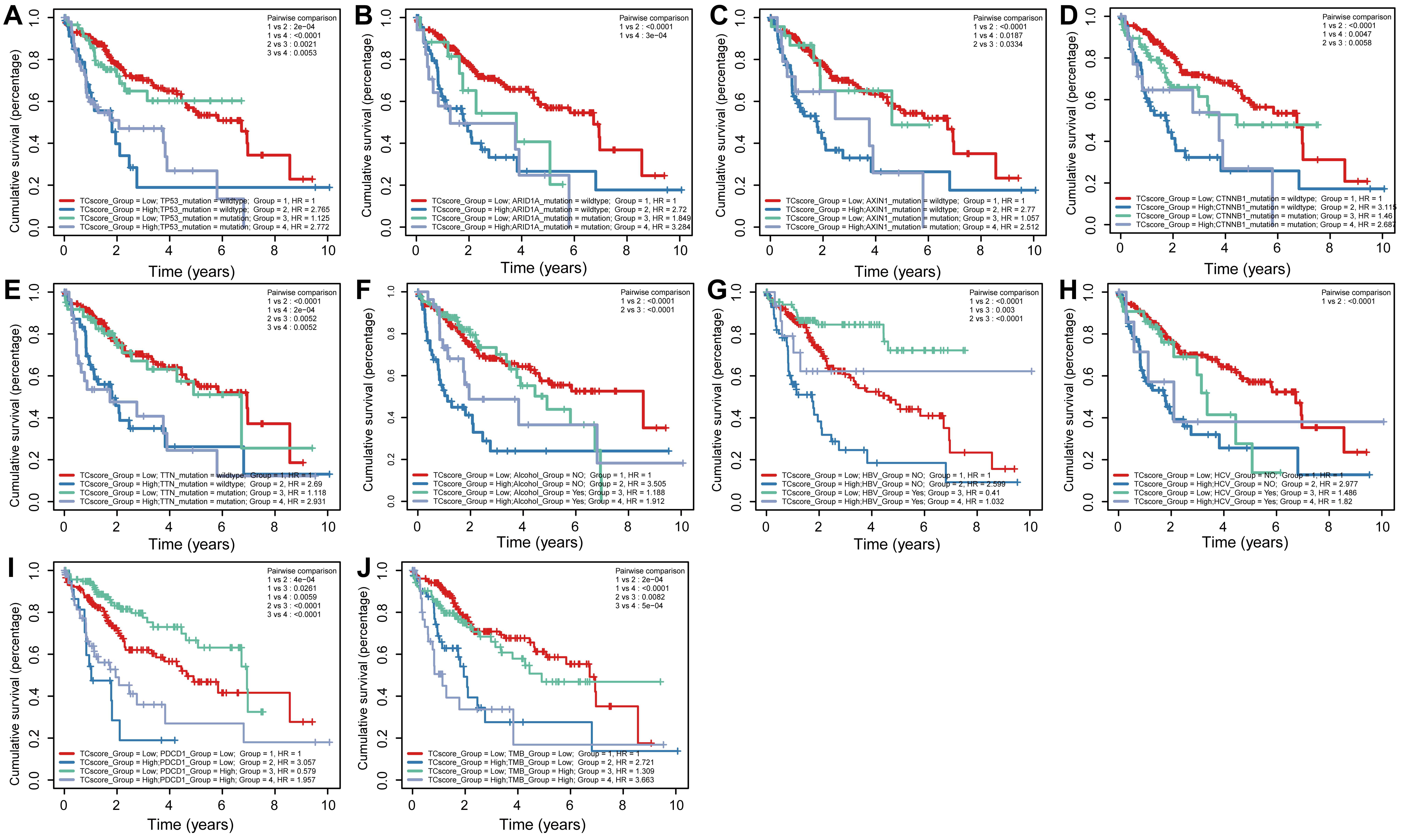

Supplement: Supplementary Figure 11 — Survival analysis using the TCscore combined with other factors. (A–J). Kaplan–Meier survival analyses were performed based on the TCscore plus the following factors: mutation statuses of (A) TP53, (B) ARID1A, (C) AXIN1, (D) CTNNB1, and (E) TTN, (F) history of alcoholism, (G) hepatitis B virus infection, (H) hepatitis C virus infection, (I) PDCD1 expression, and (J) tumor mutation burden. [file Image_11.tif]
