# Supplementary material for: Molecular Characteristics of T Cell-Mediated Tumor Killing in Hepatocellular Carcinoma
Source: Front Immunol. 2022 Apr 29;13:868480. doi: 10.3389/fimmu.2022.868480 (PMC9100886; doi:10.3389/fimmu.2022.868480)
Supplement: Supplementary file 13 [file Table_1.docx]

TableS1. Results of univariate cox regression analysis and differential analysis for 18 GSTTKs

| Gene | Univariate Cox Regression Analysis | | | Differential Analysis | |
| --- | --- | --- | --- | --- | --- |
|  | Hazard Ratio | 95% CI (LL-HL) | p-value | logFC | FDR |
| KIF11 | 1.441 | 1.191-1.744 | <0.001 | 2.107 | <0.001 |
| H2AFZ | 1.427 | 1.143-1.782 | 0.002 | 1.106 | <0.001 |
| RFPL4B | 1.267 | 1.091-1.472 | 0.002 | 7.804 | <0.001 |
| AURKA | 1.230 | 1.039-1.457 | 0.016 | 2.633 | <0.001 |
| FGF12 | 0.827 | 0.700-0.978 | 0.026 | 1.916 | <0.001 |
| NR4A3 | 0.802 | 0.662-0.971 | 0.024 | -2.241 | <0.001 |
| E2F1 | 1.184 | 1.039-1.350 | 0.011 | 3.628 | <0.001 |
| SLC1A7 | 1.151 | 1.047-1.266 | 0.004 | 1.600 | <0.001 |
| GRM4 | 1.751 | 1.262-2.429 | 0.001 | 2.723 | <0.001 |
| TGIF2LX | 1.490 | 1.001-2.216 | 0.049 | 3.810 | <0.001 |
| CDC7 | 1.472 | 1.181-1.834 | 0.001 | 1.986 | <0.001 |
| MYO1B | 0.760 | 0.632-0.913 | 0.003 | -1.165 | <0.001 |
| CA9 | 1.128 | 1.041-1.221 | 0.003 | 1.560 | <0.001 |
| CAPN11 | 0.598 | 0.372-0.960 | 0.033 | 2.105 | <0.001 |
| MCM10 | 1.549 | 1.288-1.863 | <0.001 | 2.951 | <0.001 |
| SLC4A10 | 0.387 | 0.223-0.674 | 0.001 | -1.304 | <0.001 |
| CENPF | 1.260 | 1.0937-1.451 | 0.001 | 3.798 | <0.001 |
| RECQL4 | 1.222 | 1.064-1.404 | 0.005 | 2.644 | <0.001 |
