# Supplementary material for: Molecular Characteristics of T Cell-Mediated Tumor Killing in Hepatocellular Carcinoma
Source: Front Immunol. 2022 Apr 29;13:868480. doi: 10.3389/fimmu.2022.868480 (PMC9100886; doi:10.3389/fimmu.2022.868480)
Supplement: Supplementary file 14 [file Table_2.docx]

Table S2. Molecular subtypes corresponding to each sample in the TCGA-LIHC cohort

| ID | Metabolic_Cluster | Sensitive_Cluster | Immune_Subtype |
| --- | --- | --- | --- |
| TCGA-2Y-A9GT-01A | Quiescent | Cluster2 | TGF-beta Dominant (Immune C6) |
| TCGA-2Y-A9GU-01A | Quiescent | Cluster2 | Inflammatory (Immune C3) |
| TCGA-2Y-A9GV-01A | Quiescent | Cluster2 | Inflammatory (Immune C3) |
| TCGA-2Y-A9GX-01A | Quiescent | Cluster2 | Inflammatory (Immune C3) |
| TCGA-2Y-A9H6-01A | Quiescent | Cluster2 | Inflammatory (Immune C3) |
| TCGA-4R-AA8I-01A | Quiescent | Cluster2 | Lymphocyte Depleted (Immune C4) |
| TCGA-5C-A9VH-01A | Quiescent | Cluster2 | Lymphocyte Depleted (Immune C4) |
| TCGA-5R-AA1C-01A | Quiescent | Cluster2 | Lymphocyte Depleted (Immune C4) |
| TCGA-BC-A10R-01A | Quiescent | Cluster2 | Inflammatory (Immune C3) |
| TCGA-BC-A10S-01A | Quiescent | Cluster2 | Inflammatory (Immune C3) |
| TCGA-BC-A10X-01A | Quiescent | Cluster2 | Inflammatory (Immune C3) |
| TCGA-BC-A110-01A | Quiescent | Cluster2 | Inflammatory (Immune C3) |
| TCGA-BC-A216-01A | Quiescent | Cluster1 | Wound Healing (Immune C1) |
| TCGA-BC-A217-01A | Quiescent | Cluster1 | IFN-gamma Dominant (Immune C2) |
| TCGA-BD-A2L6-01A | Quiescent | Cluster1 | Lymphocyte Depleted (Immune C4) |
| TCGA-BD-A3EP-01A | Quiescent | Cluster2 | Inflammatory (Immune C3) |
| TCGA-CC-5261-01A | Quiescent | Cluster1 | Inflammatory (Immune C3) |
| TCGA-CC-A7IK-01A | Quiescent | Cluster1 | Lymphocyte Depleted (Immune C4) |
| TCGA-CC-A8HS-01A | Quiescent | Cluster1 | Inflammatory (Immune C3) |
| TCGA-CC-A8HV-01A | Quiescent | Cluster1 | Lymphocyte Depleted (Immune C4) |
| TCGA-CC-A9FS-01A | Quiescent | Cluster1 | Inflammatory (Immune C3) |
| TCGA-DD-A115-01A | Quiescent | Cluster1 | Inflammatory (Immune C3) |
| TCGA-DD-A11B-01A | Quiescent | Cluster2 | Inflammatory (Immune C3) |
| TCGA-DD-A11C-01A | Quiescent | Cluster1 | Inflammatory (Immune C3) |
| TCGA-DD-A1EA-01A | Quiescent | Cluster2 | Inflammatory (Immune C3) |
| TCGA-DD-A1ED-01A | Quiescent | Cluster2 | Inflammatory (Immune C3) |
| TCGA-DD-A1EF-01A | Quiescent | Cluster2 | Inflammatory (Immune C3) |
| TCGA-DD-A1EH-01A | Quiescent | Cluster2 | Inflammatory (Immune C3) |
| TCGA-DD-A1EI-01A | Quiescent | Cluster2 | Wound Healing (Immune C1) |
| TCGA-DD-A3A4-01A | Quiescent | Cluster2 | Lymphocyte Depleted (Immune C4) |
| TCGA-DD-A4NG-01A | Quiescent | Cluster2 | Inflammatory (Immune C3) |
| TCGA-DD-A4NI-01A | Quiescent | Cluster2 | Inflammatory (Immune C3) |
| TCGA-DD-A4NJ-01A | Quiescent | Cluster1 | Inflammatory (Immune C3) |
| TCGA-DD-A4NP-01A | Quiescent | Cluster2 | Lymphocyte Depleted (Immune C4) |
| TCGA-DD-A4NS-01A | Quiescent | Cluster2 | Inflammatory (Immune C3) |
| TCGA-DD-AACF-01A | Quiescent | Cluster1 | Lymphocyte Depleted (Immune C4) |
| TCGA-DD-AACN-01A | Quiescent | Cluster2 | Inflammatory (Immune C3) |
| TCGA-DD-AACO-01A | Quiescent | Cluster2 | Lymphocyte Depleted (Immune C4) |
| TCGA-DD-AACQ-01A | Quiescent | Cluster1 | Lymphocyte Depleted (Immune C4) |
| TCGA-DD-AACS-01A | Quiescent | Cluster1 | Lymphocyte Depleted (Immune C4) |
| TCGA-DD-AACT-01A | Quiescent | Cluster2 | Inflammatory (Immune C3) |
| TCGA-DD-AAD1-01A | Quiescent | Cluster2 | Inflammatory (Immune C3) |
| TCGA-DD-AAD3-01A | Quiescent | Cluster2 | Inflammatory (Immune C3) |
| TCGA-DD-AAD8-01A | Quiescent | Cluster1 | Lymphocyte Depleted (Immune C4) |
| TCGA-DD-AADA-01A | Quiescent | Cluster2 | Inflammatory (Immune C3) |
| TCGA-DD-AADI-01A | Quiescent | Cluster2 | IFN-gamma Dominant (Immune C2) |
| TCGA-DD-AADQ-01A | Quiescent | Cluster2 | Lymphocyte Depleted (Immune C4) |
| TCGA-DD-AADR-01A | Quiescent | Cluster1 | Lymphocyte Depleted (Immune C4) |
| TCGA-DD-AADV-01A | Quiescent | Cluster1 | Lymphocyte Depleted (Immune C4) |
| TCGA-DD-AADY-01A | Quiescent | Cluster2 | Inflammatory (Immune C3) |
| TCGA-DD-AAE2-01A | Quiescent | Cluster2 | Inflammatory (Immune C3) |
| TCGA-DD-AAE7-01A | Quiescent | Cluster2 | Inflammatory (Immune C3) |
| TCGA-DD-AAE9-01A | Quiescent | Cluster1 | Lymphocyte Depleted (Immune C4) |
| TCGA-DD-AAEH-01A | Quiescent | Cluster2 | Inflammatory (Immune C3) |
| TCGA-DD-AAVS-01A | Quiescent | Cluster2 | Inflammatory (Immune C3) |
| TCGA-DD-AAVW-01A | Quiescent | Cluster2 | Inflammatory (Immune C3) |
| TCGA-DD-AAVX-01A | Quiescent | Cluster2 | Lymphocyte Depleted (Immune C4) |
| TCGA-DD-AAW2-01A | Quiescent | Cluster2 | Lymphocyte Depleted (Immune C4) |
| TCGA-ED-A4XI-01A | Quiescent | Cluster2 | Lymphocyte Depleted (Immune C4) |
| TCGA-ED-A5KG-01A | Quiescent | Cluster2 | IFN-gamma Dominant (Immune C2) |
| TCGA-ED-A627-01A | Quiescent | Cluster2 | Inflammatory (Immune C3) |
| TCGA-ED-A66Y-01A | Quiescent | Cluster1 | Lymphocyte Depleted (Immune C4) |
| TCGA-ED-A7PY-01A | Quiescent | Cluster2 | Inflammatory (Immune C3) |
| TCGA-ED-A7XP-01A | Quiescent | Cluster2 | Lymphocyte Depleted (Immune C4) |
| TCGA-ED-A8O5-01A | Quiescent | Cluster2 | Inflammatory (Immune C3) |
| TCGA-EP-A26S-01A | Quiescent | Cluster2 | Lymphocyte Depleted (Immune C4) |
| TCGA-FV-A23B-01A | Quiescent | Cluster1 | Inflammatory (Immune C3) |
| TCGA-FV-A3R3-01A | Quiescent | Cluster2 | Inflammatory (Immune C3) |
| TCGA-G3-A3CK-01A | Quiescent | Cluster2 | Lymphocyte Depleted (Immune C4) |
| TCGA-G3-A5SI-01A | Quiescent | Cluster1 | Lymphocyte Depleted (Immune C4) |
| TCGA-G3-A5SK-01A | Quiescent | Cluster2 | Lymphocyte Depleted (Immune C4) |
| TCGA-G3-A7M6-01A | Quiescent | Cluster1 | Inflammatory (Immune C3) |
| TCGA-G3-A7M8-01A | Quiescent | Cluster2 | Lymphocyte Depleted (Immune C4) |
| TCGA-G3-AAV0-01A | Quiescent | Cluster2 | Inflammatory (Immune C3) |
| TCGA-G3-AAV1-01A | Quiescent | Cluster2 | Lymphocyte Depleted (Immune C4) |
| TCGA-G3-AAV2-01A | Quiescent | Cluster2 | Lymphocyte Depleted (Immune C4) |
| TCGA-G3-AAV3-01A | Quiescent | Cluster2 | Inflammatory (Immune C3) |
| TCGA-HP-A5N0-01A | Quiescent | Cluster2 | Inflammatory (Immune C3) |
| TCGA-K7-A5RF-01A | Quiescent | Cluster2 | Lymphocyte Depleted (Immune C4) |
| TCGA-KR-A7K0-01A | Quiescent | Cluster2 | Lymphocyte Depleted (Immune C4) |
| TCGA-LG-A6GG-01A | Quiescent | Cluster2 | Inflammatory (Immune C3) |
| TCGA-MI-A75H-01A | Quiescent | Cluster2 | Lymphocyte Depleted (Immune C4) |
| TCGA-NI-A4U2-01A | Quiescent | Cluster2 | Lymphocyte Depleted (Immune C4) |
| TCGA-RC-A7SF-01A | Quiescent | Cluster1 | Inflammatory (Immune C3) |
| TCGA-RC-A7SH-01A | Quiescent | Cluster1 | Lymphocyte Depleted (Immune C4) |
| TCGA-RC-A7SK-01A | Quiescent | Cluster2 | Lymphocyte Depleted (Immune C4) |
| TCGA-UB-A7MC-01A | Quiescent | Cluster1 | Lymphocyte Depleted (Immune C4) |
| TCGA-UB-AA0U-01A | Quiescent | Cluster1 | Inflammatory (Immune C3) |
| TCGA-UB-AA0V-01A | Quiescent | Cluster2 | Inflammatory (Immune C3) |
| TCGA-WX-AA44-01A | Quiescent | Cluster1 | Inflammatory (Immune C3) |
| TCGA-WX-AA46-01A | Quiescent | Cluster2 | Inflammatory (Immune C3) |
| TCGA-XR-A8TF-01A | Quiescent | Cluster2 | Inflammatory (Immune C3) |
| TCGA-ZP-A9CV-01A | Quiescent | Cluster2 | Lymphocyte Depleted (Immune C4) |
| TCGA-ZP-A9CY-01A | Quiescent | Cluster2 | Lymphocyte Depleted (Immune C4) |
| TCGA-ZP-A9D0-01A | Quiescent | Cluster2 | Inflammatory (Immune C3) |
| TCGA-ZP-A9D1-01A | Quiescent | Cluster2 | Inflammatory (Immune C3) |
| TCGA-ZP-A9D2-01A | Quiescent | Cluster2 | IFN-gamma Dominant (Immune C2) |
| TCGA-ZP-A9D4-01A | Quiescent | Cluster2 | Lymphocyte Depleted (Immune C4) |
| TCGA-2Y-A9GS-01A | Glycolytic | Cluster1 | Lymphocyte Depleted (Immune C4) |
| TCGA-2Y-A9GW-01A | Glycolytic | Cluster2 | Inflammatory (Immune C3) |
| TCGA-2Y-A9GY-01A | Glycolytic | Cluster1 | IFN-gamma Dominant (Immune C2) |
| TCGA-2Y-A9H0-01A | Glycolytic | Cluster1 | IFN-gamma Dominant (Immune C2) |
| TCGA-2Y-A9H2-01A | Glycolytic | Cluster1 | Inflammatory (Immune C3) |
| TCGA-2Y-A9H3-01A | Glycolytic | Cluster2 | Inflammatory (Immune C3) |
| TCGA-5C-A9VG-01A | Glycolytic | Cluster1 | Lymphocyte Depleted (Immune C4) |
| TCGA-5R-AA1D-01A | Glycolytic | Cluster2 | Inflammatory (Immune C3) |
| TCGA-BC-A10U-01A | Glycolytic | Cluster2 | Inflammatory (Immune C3) |
| TCGA-BC-A10W-01A | Glycolytic | Cluster1 | Lymphocyte Depleted (Immune C4) |
| TCGA-BC-A112-01A | Glycolytic | Cluster1 | Lymphocyte Depleted (Immune C4) |
| TCGA-BC-A8YO-01A | Glycolytic | Cluster1 | Lymphocyte Depleted (Immune C4) |
| TCGA-CC-5260-01A | Glycolytic | Cluster1 | Inflammatory (Immune C3) |
| TCGA-CC-5263-01A | Glycolytic | Cluster1 | Lymphocyte Depleted (Immune C4) |
| TCGA-CC-A1HT-01A | Glycolytic | Cluster1 | IFN-gamma Dominant (Immune C2) |
| TCGA-CC-A3M9-01A | Glycolytic | Cluster1 | IFN-gamma Dominant (Immune C2) |
| TCGA-CC-A3MA-01A | Glycolytic | Cluster1 | Wound Healing (Immune C1) |
| TCGA-CC-A3MC-01A | Glycolytic | Cluster1 | Inflammatory (Immune C3) |
| TCGA-CC-A7IE-01A | Glycolytic | Cluster1 | IFN-gamma Dominant (Immune C2) |
| TCGA-CC-A7II-01A | Glycolytic | Cluster1 | Wound Healing (Immune C1) |
| TCGA-CC-A7IJ-01A | Glycolytic | Cluster1 | IFN-gamma Dominant (Immune C2) |
| TCGA-CC-A8HT-01A | Glycolytic | Cluster1 | Lymphocyte Depleted (Immune C4) |
| TCGA-CC-A8HU-01A | Glycolytic | Cluster1 | Lymphocyte Depleted (Immune C4) |
| TCGA-DD-A113-01A | Glycolytic | Cluster1 | Inflammatory (Immune C3) |
| TCGA-DD-A114-01A | Glycolytic | Cluster1 | IFN-gamma Dominant (Immune C2) |
| TCGA-DD-A118-01A | Glycolytic | Cluster2 | Inflammatory (Immune C3) |
| TCGA-DD-A1EC-01A | Glycolytic | Cluster1 | Inflammatory (Immune C3) |
| TCGA-DD-A1EL-01A | Glycolytic | Cluster1 | Lymphocyte Depleted (Immune C4) |
| TCGA-DD-A39V-01A | Glycolytic | Cluster2 | IFN-gamma Dominant (Immune C2) |
| TCGA-DD-A3A7-01A | Glycolytic | Cluster1 | Inflammatory (Immune C3) |
| TCGA-DD-A3A9-01A | Glycolytic | Cluster2 | Inflammatory (Immune C3) |
| TCGA-DD-A4NA-01A | Glycolytic | Cluster2 | Inflammatory (Immune C3) |
| TCGA-DD-A4NB-01A | Glycolytic | Cluster2 | Inflammatory (Immune C3) |
| TCGA-DD-A4NH-01A | Glycolytic | Cluster1 | Inflammatory (Immune C3) |
| TCGA-DD-AACH-01A | Glycolytic | Cluster1 | Lymphocyte Depleted (Immune C4) |
| TCGA-DD-AACI-01A | Glycolytic | Cluster2 | Inflammatory (Immune C3) |
| TCGA-DD-AACL-01A | Glycolytic | Cluster1 | IFN-gamma Dominant (Immune C2) |
| TCGA-DD-AACX-01A | Glycolytic | Cluster1 | Lymphocyte Depleted (Immune C4) |
| TCGA-DD-AAD2-01A | Glycolytic | Cluster2 | Inflammatory (Immune C3) |
| TCGA-DD-AAD5-01A | Glycolytic | Cluster1 | Lymphocyte Depleted (Immune C4) |
| TCGA-DD-AAE0-01A | Glycolytic | Cluster1 | Wound Healing (Immune C1) |
| TCGA-DD-AAVR-01A | Glycolytic | Cluster2 | Inflammatory (Immune C3) |
| TCGA-DD-AAVU-01A | Glycolytic | Cluster2 | Lymphocyte Depleted (Immune C4) |
| TCGA-DD-AAVV-01A | Glycolytic | Cluster2 | Inflammatory (Immune C3) |
| TCGA-ED-A459-01A | Glycolytic | Cluster1 | Lymphocyte Depleted (Immune C4) |
| TCGA-ED-A7PX-01A | Glycolytic | Cluster2 | Wound Healing (Immune C1) |
| TCGA-ED-A7XO-01A | Glycolytic | Cluster1 | IFN-gamma Dominant (Immune C2) |
| TCGA-ED-A82E-01A | Glycolytic | Cluster2 | Inflammatory (Immune C3) |
| TCGA-EP-A2KA-01A | Glycolytic | Cluster1 | IFN-gamma Dominant (Immune C2) |
| TCGA-G3-A25S-01A | Glycolytic | Cluster1 | Lymphocyte Depleted (Immune C4) |
| TCGA-G3-A25T-01A | Glycolytic | Cluster1 | Wound Healing (Immune C1) |
| TCGA-G3-A25V-01A | Glycolytic | Cluster2 | Inflammatory (Immune C3) |
| TCGA-G3-A25X-01A | Glycolytic | Cluster1 | IFN-gamma Dominant (Immune C2) |
| TCGA-G3-A25Y-01A | Glycolytic | Cluster1 | IFN-gamma Dominant (Immune C2) |
| TCGA-G3-A5SJ-01A | Glycolytic | Cluster1 | Wound Healing (Immune C1) |
| TCGA-G3-A5SM-01A | Glycolytic | Cluster2 | Inflammatory (Immune C3) |
| TCGA-G3-A7M7-01A | Glycolytic | Cluster2 | Lymphocyte Depleted (Immune C4) |
| TCGA-G3-A7M9-01A | Glycolytic | Cluster1 | Wound Healing (Immune C1) |
| TCGA-G3-AAV7-01A | Glycolytic | Cluster1 | Lymphocyte Depleted (Immune C4) |
| TCGA-GJ-A3OU-01A | Glycolytic | Cluster2 | Inflammatory (Immune C3) |
| TCGA-K7-A5RG-01A | Glycolytic | Cluster1 | IFN-gamma Dominant (Immune C2) |
| TCGA-MI-A75C-01A | Glycolytic | Cluster2 | Lymphocyte Depleted (Immune C4) |
| TCGA-MI-A75I-01A | Glycolytic | Cluster1 | Lymphocyte Depleted (Immune C4) |
| TCGA-MR-A8JO-01A | Glycolytic | Cluster2 | Inflammatory (Immune C3) |
| TCGA-PD-A5DF-01A | Glycolytic | Cluster1 | Lymphocyte Depleted (Immune C4) |
| TCGA-RC-A6M5-01A | Glycolytic | Cluster2 | Inflammatory (Immune C3) |
| TCGA-RC-A6M6-01A | Glycolytic | Cluster1 | Wound Healing (Immune C1) |
| TCGA-RC-A7S9-01A | Glycolytic | Cluster1 | Lymphocyte Depleted (Immune C4) |
| TCGA-UB-A7MD-01A | Glycolytic | Cluster2 | Lymphocyte Depleted (Immune C4) |
| TCGA-WQ-A9G7-01A | Glycolytic | Cluster1 | Wound Healing (Immune C1) |
| TCGA-WX-AA47-01A | Glycolytic | Cluster2 | Lymphocyte Depleted (Immune C4) |
| TCGA-YA-A8S7-01A | Glycolytic | Cluster1 | Lymphocyte Depleted (Immune C4) |
| TCGA-2Y-A9GZ-01A | Cholesterogenic | Cluster2 | Inflammatory (Immune C3) |
| TCGA-2Y-A9H1-01A | Cholesterogenic | Cluster2 | Lymphocyte Depleted (Immune C4) |
| TCGA-2Y-A9H4-01A | Cholesterogenic | Cluster2 | Lymphocyte Depleted (Immune C4) |
| TCGA-2Y-A9H5-01A | Cholesterogenic | Cluster2 | Inflammatory (Immune C3) |
| TCGA-2Y-A9H7-01A | Cholesterogenic | Cluster2 | Inflammatory (Immune C3) |
| TCGA-2Y-A9H8-01A | Cholesterogenic | Cluster1 | IFN-gamma Dominant (Immune C2) |
| TCGA-2Y-A9H9-01A | Cholesterogenic | Cluster2 | Lymphocyte Depleted (Immune C4) |
| TCGA-2Y-A9HA-01A | Cholesterogenic | Cluster2 | Lymphocyte Depleted (Immune C4) |
| TCGA-2Y-A9HB-01A | Cholesterogenic | Cluster2 | Lymphocyte Depleted (Immune C4) |
| TCGA-3K-AAZ8-01A | Cholesterogenic | Cluster2 | Lymphocyte Depleted (Immune C4) |
| TCGA-5R-AAAM-01A | Cholesterogenic | Cluster2 | Inflammatory (Immune C3) |
| TCGA-BC-A10Y-01A | Cholesterogenic | Cluster1 | Lymphocyte Depleted (Immune C4) |
| TCGA-BC-A10Z-01A | Cholesterogenic | Cluster2 | Lymphocyte Depleted (Immune C4) |
| TCGA-BC-A3KF-01A | Cholesterogenic | Cluster2 | Inflammatory (Immune C3) |
| TCGA-BC-A3KG-01A | Cholesterogenic | Cluster1 | Lymphocyte Depleted (Immune C4) |
| TCGA-BC-A5W4-01A | Cholesterogenic | Cluster2 | Lymphocyte Depleted (Immune C4) |
| TCGA-BC-A69I-01A | Cholesterogenic | Cluster2 | Inflammatory (Immune C3) |
| TCGA-BW-A5NO-01A | Cholesterogenic | Cluster2 | Lymphocyte Depleted (Immune C4) |
| TCGA-CC-A5UE-01A | Cholesterogenic | Cluster1 | Lymphocyte Depleted (Immune C4) |
| TCGA-CC-A7IF-01A | Cholesterogenic | Cluster1 | Lymphocyte Depleted (Immune C4) |
| TCGA-CC-A7IH-01A | Cholesterogenic | Cluster2 | Inflammatory (Immune C3) |
| TCGA-CC-A7IL-01A | Cholesterogenic | Cluster2 | Lymphocyte Depleted (Immune C4) |
| TCGA-DD-A119-01A | Cholesterogenic | Cluster2 | Lymphocyte Depleted (Immune C4) |
| TCGA-DD-A11D-01A | Cholesterogenic | Cluster2 | Lymphocyte Depleted (Immune C4) |
| TCGA-DD-A1EB-01A | Cholesterogenic | Cluster2 | Lymphocyte Depleted (Immune C4) |
| TCGA-DD-A1EE-01A | Cholesterogenic | Cluster2 | Lymphocyte Depleted (Immune C4) |
| TCGA-DD-A39W-01A | Cholesterogenic | Cluster2 | Inflammatory (Immune C3) |
| TCGA-DD-A39Z-01A | Cholesterogenic | Cluster1 | Lymphocyte Depleted (Immune C4) |
| TCGA-DD-A3A2-01A | Cholesterogenic | Cluster2 | Inflammatory (Immune C3) |
| TCGA-DD-A3A5-01A | Cholesterogenic | Cluster2 | Lymphocyte Depleted (Immune C4) |
| TCGA-DD-A3A8-01A | Cholesterogenic | Cluster2 | Lymphocyte Depleted (Immune C4) |
| TCGA-DD-A4ND-01A | Cholesterogenic | Cluster2 | Inflammatory (Immune C3) |
| TCGA-DD-A4NE-01A | Cholesterogenic | Cluster1 | Lymphocyte Depleted (Immune C4) |
| TCGA-DD-A4NF-01A | Cholesterogenic | Cluster2 | Lymphocyte Depleted (Immune C4) |
| TCGA-DD-A4NK-01A | Cholesterogenic | Cluster2 | Inflammatory (Immune C3) |
| TCGA-DD-A4NL-01A | Cholesterogenic | Cluster2 | Inflammatory (Immune C3) |
| TCGA-DD-A4NN-01A | Cholesterogenic | Cluster1 | Inflammatory (Immune C3) |
| TCGA-DD-A4NO-01A | Cholesterogenic | Cluster2 | Inflammatory (Immune C3) |
| TCGA-DD-A4NV-01A | Cholesterogenic | Cluster2 | Inflammatory (Immune C3) |
| TCGA-DD-A73A-01A | Cholesterogenic | Cluster2 | Inflammatory (Immune C3) |
| TCGA-DD-A73C-01A | Cholesterogenic | Cluster2 | Inflammatory (Immune C3) |
| TCGA-DD-A73D-01A | Cholesterogenic | Cluster2 | Lymphocyte Depleted (Immune C4) |
| TCGA-DD-A73E-01A | Cholesterogenic | Cluster2 | Inflammatory (Immune C3) |
| TCGA-DD-A73F-01A | Cholesterogenic | Cluster1 | IFN-gamma Dominant (Immune C2) |
| TCGA-DD-AAC9-01A | Cholesterogenic | Cluster2 | Inflammatory (Immune C3) |
| TCGA-DD-AACA-01A | Cholesterogenic | Cluster1 | Inflammatory (Immune C3) |
| TCGA-DD-AACD-01A | Cholesterogenic | Cluster2 | Lymphocyte Depleted (Immune C4) |
| TCGA-DD-AACE-01A | Cholesterogenic | Cluster2 | Lymphocyte Depleted (Immune C4) |
| TCGA-DD-AACK-01A | Cholesterogenic | Cluster2 | Lymphocyte Depleted (Immune C4) |
| TCGA-DD-AACU-01A | Cholesterogenic | Cluster2 | Lymphocyte Depleted (Immune C4) |
| TCGA-DD-AACV-01A | Cholesterogenic | Cluster1 | Lymphocyte Depleted (Immune C4) |
| TCGA-DD-AACW-01A | Cholesterogenic | Cluster1 | Lymphocyte Depleted (Immune C4) |
| TCGA-DD-AACY-01A | Cholesterogenic | Cluster2 | Lymphocyte Depleted (Immune C4) |
| TCGA-DD-AAD6-01A | Cholesterogenic | Cluster1 | Lymphocyte Depleted (Immune C4) |
| TCGA-DD-AADB-01A | Cholesterogenic | Cluster1 | IFN-gamma Dominant (Immune C2) |
| TCGA-DD-AADC-01A | Cholesterogenic | Cluster1 | Lymphocyte Depleted (Immune C4) |
| TCGA-DD-AADD-01A | Cholesterogenic | Cluster1 | Lymphocyte Depleted (Immune C4) |
| TCGA-DD-AADF-01A | Cholesterogenic | Cluster1 | Lymphocyte Depleted (Immune C4) |
| TCGA-DD-AADG-01A | Cholesterogenic | Cluster2 | Lymphocyte Depleted (Immune C4) |
| TCGA-DD-AADJ-01A | Cholesterogenic | Cluster2 | Inflammatory (Immune C3) |
| TCGA-DD-AADK-01A | Cholesterogenic | Cluster2 | Inflammatory (Immune C3) |
| TCGA-DD-AADL-01A | Cholesterogenic | Cluster1 | Lymphocyte Depleted (Immune C4) |
| TCGA-DD-AADP-01A | Cholesterogenic | Cluster2 | Inflammatory (Immune C3) |
| TCGA-DD-AADS-01A | Cholesterogenic | Cluster2 | Lymphocyte Depleted (Immune C4) |
| TCGA-DD-AADU-01A | Cholesterogenic | Cluster2 | Inflammatory (Immune C3) |
| TCGA-DD-AADW-01A | Cholesterogenic | Cluster1 | Wound Healing (Immune C1) |
| TCGA-DD-AAE1-01A | Cholesterogenic | Cluster2 | Lymphocyte Depleted (Immune C4) |
| TCGA-DD-AAE3-01A | Cholesterogenic | Cluster2 | Lymphocyte Depleted (Immune C4) |
| TCGA-DD-AAE4-01A | Cholesterogenic | Cluster1 | IFN-gamma Dominant (Immune C2) |
| TCGA-DD-AAEA-01A | Cholesterogenic | Cluster1 | Lymphocyte Depleted (Immune C4) |
| TCGA-DD-AAEB-01A | Cholesterogenic | Cluster2 | Inflammatory (Immune C3) |
| TCGA-DD-AAED-01A | Cholesterogenic | Cluster2 | Lymphocyte Depleted (Immune C4) |
| TCGA-DD-AAEE-01A | Cholesterogenic | Cluster2 | Lymphocyte Depleted (Immune C4) |
| TCGA-DD-AAEG-01A | Cholesterogenic | Cluster2 | Lymphocyte Depleted (Immune C4) |
| TCGA-DD-AAEK-01A | Cholesterogenic | Cluster2 | Inflammatory (Immune C3) |
| TCGA-DD-AAVQ-01A | Cholesterogenic | Cluster1 | IFN-gamma Dominant (Immune C2) |
| TCGA-DD-AAVY-01A | Cholesterogenic | Cluster2 | Lymphocyte Depleted (Immune C4) |
| TCGA-DD-AAVZ-01A | Cholesterogenic | Cluster2 | Lymphocyte Depleted (Immune C4) |
| TCGA-DD-AAW0-01A | Cholesterogenic | Cluster2 | Inflammatory (Immune C3) |
| TCGA-DD-AAW1-01A | Cholesterogenic | Cluster2 | Lymphocyte Depleted (Immune C4) |
| TCGA-DD-AAW3-01A | Cholesterogenic | Cluster2 | Lymphocyte Depleted (Immune C4) |
| TCGA-ED-A66X-01A | Cholesterogenic | Cluster1 | Inflammatory (Immune C3) |
| TCGA-ED-A8O6-01A | Cholesterogenic | Cluster1 | Inflammatory (Immune C3) |
| TCGA-EP-A12J-01A | Cholesterogenic | Cluster2 | Lymphocyte Depleted (Immune C4) |
| TCGA-EP-A2KB-01A | Cholesterogenic | Cluster1 | Inflammatory (Immune C3) |
| TCGA-EP-A2KC-01A | Cholesterogenic | Cluster2 | Lymphocyte Depleted (Immune C4) |
| TCGA-ES-A2HS-01A | Cholesterogenic | Cluster2 | Inflammatory (Immune C3) |
| TCGA-FV-A2QR-01A | Cholesterogenic | Cluster2 | Inflammatory (Immune C3) |
| TCGA-FV-A3R2-01A | Cholesterogenic | Cluster2 | Lymphocyte Depleted (Immune C4) |
| TCGA-FV-A495-01A | Cholesterogenic | Cluster1 | IFN-gamma Dominant (Immune C2) |
| TCGA-FV-A496-01A | Cholesterogenic | Cluster2 | Inflammatory (Immune C3) |
| TCGA-G3-A25U-01A | Cholesterogenic | Cluster1 | Lymphocyte Depleted (Immune C4) |
| TCGA-G3-A3CH-01A | Cholesterogenic | Cluster1 | Inflammatory (Immune C3) |
| TCGA-G3-A3CI-01A | Cholesterogenic | Cluster2 | Inflammatory (Immune C3) |
| TCGA-G3-A3CJ-01A | Cholesterogenic | Cluster2 | Lymphocyte Depleted (Immune C4) |
| TCGA-G3-A5SL-01A | Cholesterogenic | Cluster2 | Inflammatory (Immune C3) |
| TCGA-G3-A6UC-01A | Cholesterogenic | Cluster2 | Lymphocyte Depleted (Immune C4) |
| TCGA-G3-A7M5-01A | Cholesterogenic | Cluster2 | Lymphocyte Depleted (Immune C4) |
| TCGA-G3-AAUZ-01A | Cholesterogenic | Cluster1 | IFN-gamma Dominant (Immune C2) |
| TCGA-G3-AAV4-01A | Cholesterogenic | Cluster2 | Lymphocyte Depleted (Immune C4) |
| TCGA-GJ-A9DB-01A | Cholesterogenic | Cluster2 | Inflammatory (Immune C3) |
| TCGA-K7-A6G5-01A | Cholesterogenic | Cluster2 | Lymphocyte Depleted (Immune C4) |
| TCGA-LG-A9QC-01A | Cholesterogenic | Cluster2 | Lymphocyte Depleted (Immune C4) |
| TCGA-LG-A9QD-01A | Cholesterogenic | Cluster2 | Lymphocyte Depleted (Immune C4) |
| TCGA-MI-A75G-01A | Cholesterogenic | Cluster2 | Lymphocyte Depleted (Immune C4) |
| TCGA-MR-A520-01A | Cholesterogenic | Cluster2 | Lymphocyte Depleted (Immune C4) |
| TCGA-NI-A8LF-01A | Cholesterogenic | Cluster2 | Inflammatory (Immune C3) |
| TCGA-O8-A75V-01A | Cholesterogenic | Cluster2 | Inflammatory (Immune C3) |
| TCGA-RC-A6M4-01A | Cholesterogenic | Cluster1 | Lymphocyte Depleted (Immune C4) |
| TCGA-RC-A7SB-01A | Cholesterogenic | Cluster2 | Lymphocyte Depleted (Immune C4) |
| TCGA-UB-A7MB-01A | Cholesterogenic | Cluster1 | Lymphocyte Depleted (Immune C4) |
| TCGA-UB-A7ME-01A | Cholesterogenic | Cluster2 | Inflammatory (Immune C3) |
| TCGA-WJ-A86L-01A | Cholesterogenic | Cluster1 | Lymphocyte Depleted (Immune C4) |
| TCGA-WQ-AB4B-01A | Cholesterogenic | Cluster2 | Lymphocyte Depleted (Immune C4) |
| TCGA-XR-A8TC-01A | Cholesterogenic | Cluster1 | Lymphocyte Depleted (Immune C4) |
| TCGA-XR-A8TE-01A | Cholesterogenic | Cluster2 | Inflammatory (Immune C3) |
| TCGA-XR-A8TG-01A | Cholesterogenic | Cluster1 | Inflammatory (Immune C3) |
| TCGA-ZS-A9CD-01A | Cholesterogenic | Cluster2 | Inflammatory (Immune C3) |
| TCGA-ZS-A9CE-01A | Cholesterogenic | Cluster2 | Lymphocyte Depleted (Immune C4) |
| TCGA-ZS-A9CF-01A | Cholesterogenic | Cluster1 | Lymphocyte Depleted (Immune C4) |
| TCGA-ZS-A9CG-01A | Cholesterogenic | Cluster2 | Inflammatory (Immune C3) |
| TCGA-BC-A10T-01A | Mixed | Cluster2 | Wound Healing (Immune C1) |
| TCGA-BC-A69H-01A | Mixed | Cluster1 | Lymphocyte Depleted (Immune C4) |
| TCGA-CC-5258-01A | Mixed | Cluster1 | Lymphocyte Depleted (Immune C4) |
| TCGA-CC-5259-01A | Mixed | Cluster1 | Inflammatory (Immune C3) |
| TCGA-CC-5262-01A | Mixed | Cluster1 | IFN-gamma Dominant (Immune C2) |
| TCGA-CC-5264-01A | Mixed | Cluster1 | Wound Healing (Immune C1) |
| TCGA-CC-A123-01A | Mixed | Cluster2 | Lymphocyte Depleted (Immune C4) |
| TCGA-CC-A3MB-01A | Mixed | Cluster1 | Lymphocyte Depleted (Immune C4) |
| TCGA-CC-A5UC-01A | Mixed | Cluster1 | Wound Healing (Immune C1) |
| TCGA-CC-A5UD-01A | Mixed | Cluster1 | Lymphocyte Depleted (Immune C4) |
| TCGA-CC-A7IG-01A | Mixed | Cluster1 | Wound Healing (Immune C1) |
| TCGA-CC-A9FW-01A | Mixed | Cluster1 | Inflammatory (Immune C3) |
| TCGA-DD-A116-01A | Mixed | Cluster2 | Lymphocyte Depleted (Immune C4) |
| TCGA-DD-A1EG-01A | Mixed | Cluster2 | IFN-gamma Dominant (Immune C2) |
| TCGA-DD-A1EJ-01A | Mixed | Cluster1 | Lymphocyte Depleted (Immune C4) |
| TCGA-DD-A1EK-01A | Mixed | Cluster2 | Inflammatory (Immune C3) |
| TCGA-DD-A39X-01A | Mixed | Cluster2 | Inflammatory (Immune C3) |
| TCGA-DD-A39Y-01A | Mixed | Cluster1 | Lymphocyte Depleted (Immune C4) |
| TCGA-DD-A3A1-01A | Mixed | Cluster2 | Lymphocyte Depleted (Immune C4) |
| TCGA-DD-A3A3-01A | Mixed | Cluster2 | Lymphocyte Depleted (Immune C4) |
| TCGA-DD-A4NQ-01A | Mixed | Cluster1 | Lymphocyte Depleted (Immune C4) |
| TCGA-DD-A4NR-01A | Mixed | Cluster1 | IFN-gamma Dominant (Immune C2) |
| TCGA-DD-A73B-01A | Mixed | Cluster1 | Lymphocyte Depleted (Immune C4) |
| TCGA-DD-A73G-01A | Mixed | Cluster1 | Lymphocyte Depleted (Immune C4) |
| TCGA-DD-AAC8-01A | Mixed | Cluster2 | Lymphocyte Depleted (Immune C4) |
| TCGA-DD-AACB-01A | Mixed | Cluster1 | IFN-gamma Dominant (Immune C2) |
| TCGA-DD-AACC-01A | Mixed | Cluster2 | IFN-gamma Dominant (Immune C2) |
| TCGA-DD-AACG-01A | Mixed | Cluster1 | Lymphocyte Depleted (Immune C4) |
| TCGA-DD-AACJ-01A | Mixed | Cluster2 | Lymphocyte Depleted (Immune C4) |
| TCGA-DD-AACP-01A | Mixed | Cluster1 | Wound Healing (Immune C1) |
| TCGA-DD-AACZ-01A | Mixed | Cluster1 | IFN-gamma Dominant (Immune C2) |
| TCGA-DD-AAD0-01A | Mixed | Cluster1 | Lymphocyte Depleted (Immune C4) |
| TCGA-DD-AADM-01A | Mixed | Cluster1 | Lymphocyte Depleted (Immune C4) |
| TCGA-DD-AADN-01A | Mixed | Cluster1 | IFN-gamma Dominant (Immune C2) |
| TCGA-DD-AADO-01A | Mixed | Cluster2 | IFN-gamma Dominant (Immune C2) |
| TCGA-DD-AAE6-01A | Mixed | Cluster1 | Lymphocyte Depleted (Immune C4) |
| TCGA-DD-AAEI-01A | Mixed | Cluster1 | Lymphocyte Depleted (Immune C4) |
| TCGA-DD-AAVP-01A | Mixed | Cluster2 | Inflammatory (Immune C3) |
| TCGA-ED-A7PZ-01A | Mixed | Cluster1 | Lymphocyte Depleted (Immune C4) |
| TCGA-EP-A3JL-01A | Mixed | Cluster1 | IFN-gamma Dominant (Immune C2) |
| TCGA-EP-A3RK-01A | Mixed | Cluster1 | IFN-gamma Dominant (Immune C2) |
| TCGA-ES-A2HT-01A | Mixed | Cluster2 | Lymphocyte Depleted (Immune C4) |
| TCGA-FV-A2QQ-01A | Mixed | Cluster2 | Inflammatory (Immune C3) |
| TCGA-FV-A3I1-01A | Mixed | Cluster2 | Inflammatory (Immune C3) |
| TCGA-FV-A4ZP-01A | Mixed | Cluster1 | Lymphocyte Depleted (Immune C4) |
| TCGA-FV-A4ZQ-01A | Mixed | Cluster1 | IFN-gamma Dominant (Immune C2) |
| TCGA-G3-A25Z-01A | Mixed | Cluster2 | Lymphocyte Depleted (Immune C4) |
| TCGA-G3-AAV5-01A | Mixed | Cluster2 | Lymphocyte Depleted (Immune C4) |
| TCGA-G3-AAV6-01A | Mixed | Cluster1 | Wound Healing (Immune C1) |
| TCGA-HP-A5MZ-01A | Mixed | Cluster2 | Inflammatory (Immune C3) |
| TCGA-KR-A7K7-01A | Mixed | Cluster1 | IFN-gamma Dominant (Immune C2) |
| TCGA-KR-A7K8-01A | Mixed | Cluster2 | Inflammatory (Immune C3) |
| TCGA-MI-A75E-01A | Mixed | Cluster2 | Inflammatory (Immune C3) |
| TCGA-QA-A7B7-01A | Mixed | Cluster1 | Lymphocyte Depleted (Immune C4) |
| TCGA-RG-A7D4-01A | Mixed | Cluster1 | IFN-gamma Dominant (Immune C2) |
| TCGA-UB-A7MF-01A | Mixed | Cluster1 | IFN-gamma Dominant (Immune C2) |
| TCGA-XR-A8TD-01A | Mixed | Cluster1 | IFN-gamma Dominant (Immune C2) |
| TCGA-ZP-A9CZ-01A | Mixed | Cluster1 | IFN-gamma Dominant (Immune C2) |
